# Fortified relaxor ferroelectricity of rare earth substituted 4-layered BaBi_3.9_RE_0.1_Ti_4_O_15_ (RE = La, Pr, Nd, and Sm) Aurivillius compounds

**DOI:** 10.1038/s41598-022-18855-9

**Published:** 2022-10-03

**Authors:** Tirupathi Patri, Avijit Ghosh, M. L. V. Mahesh, P. D. Babu, S. K. Mandal, M. N. Singh

**Affiliations:** 1 Department of Physics, Rajiv Gandhi University of Knowledge Technologies, Srikakulam, Andhra Pradesh 532402 India; 2 Department of Physics, Central University of Jharkhand, Ranchi, Jharkhand 835205 India; 3 Defence Metallurgical Research Laboratory, Kanchan Bagh, Hyderabad, Telangana 500066 India; 4 UGC-DAE Consortium for Scientific Research, Mumbai Center, BARC, Mumbai, 400085 India; 5 Surface Physics and Materials Science Division, Saha Institute of Nuclear Physics, Kolkata, Sector-1, AF Block, Bidhannagar, Kolkata, 700064 India; 6 HXAL Synchrotrons Utilization Section, Raja Ramanna Centre for Advanced Technology, Indore, 452013 India

**Keywords:** Ferroelectrics and multiferroics, Physics, Condensed-matter physics, Ferroelectrics and multiferroics

## Abstract

In this report, the effect of rare-earth (RE^3+^) ion substitution on structural, microstructural, and electrical properties in barium bismuth titanate (BaBi_4_Ti_4_O_15_) (BBTO) Aurivillius ceramics has been investigated. The Rietveld refinements on X-ray diffraction (XRD) patterns confirm that all the samples have an orthorhombic crystal system with A2_1_am space group. Meanwhile, temperature dependent synchrotron XRD patterns reveal that the existence of dual phase in higher temperature region. The randomly oriented plate-like grains are experimentally strived to confirm the distinctive feature of bismuth layered Aurivillius ceramics. The broad band dielectric spectroscopic investigation signifies a shifting of ferroelectric phase transition (T_m_) towards low temperature region with a decrease of the RE^3+^-ionic radii in BBTO ceramics. The origin of diffuse ferroelectric phase transitions followed by stabilization of the relaxor ferroelectric nature at high frequency region is explained using suitable standard models. The temperature dependent ac and dc conductivity results indicate the presence of double ionized oxygen vacancies in BBTO ceramics, whereas the dominance of single ionized oxygen vacancies is observed in RE-substituted BBTO ceramics. The room temperature polarization vs. electric field (P–E) hysteresis loops are shown to be well-shaped symmetric for BBTO ceramics, whereas slim asymmetric ferroelectric characteristics developed at RE-substituted BBTO ceramics.

## Introduction

In the year 1949, a renowned scholar Karin Aurivillius investigated a family of bismuth based perovskite layered structures, known as Aurivillius ferroelectric oxides^1,2^. Most of the Aurivillius perovskites are ferroelectrics, which exhibit excellent ferroelectricity, large piezoelectric constant and high Curie temperature state greater than 400 °C, rather than lead-based piezoelectric materials (e.g., PbTiO_3_, PZT, PLZT etc.)^1–5^. Over the last few decades, there has been extensive research carried out, especially on bismuth-based Aurivillius phase materials of general formulae Bi_4_Ti_3_O_12_, SrBi_2_Nb_2_O_9_, and BaBi_2_Nb_2_O_9,_ which have excellent optical, dielectric and relaxor ferroelectric properties^6–10^. Hereafter, Aurivillius ferroelectric perovskite has received tremendous attention for high temperature piezoelectric devices, non-volatile random-access memories (NVRAM), transducers, sensors, etc.^5,8–10^.

In general, the bismuth based Aurivillius perovskite layers are interleaved by perovskite blocks of (A_n−1_B_n_O_3n+1_)^2−^ with fluorite-like layers (Bi_2_O_2_)^2+^, where n refers to the number of perovskite-like layers shared with BO_6_ octahedra to form the perovskite blocks. Here, A-site is filled up with mono-, di- and trivalent cations and their permutations consisted of A = Na^+^, Sr^2+^, Bi^3+^, Ba^2+^, Ca^2+^, Ln^3+^ etc., in 12-fold coordination, whereas B-site is filled up with small cations consisted of B = Ti^4+^, Nb^5+^, W^6+^, Fe^3+^, Cr^3+^ etc., in six-fold coordination, respectively^1,2,6–13^. The preliminary series of Aurivillius compounds with the chemical formula Bi_4_Ti_3_O_12_ (n = 3) and Bi_5_Ti_4_O_15_ (n = 4) are well renowned for their excellent dielectric and ferroelectric properties with a very low fatigue nature. In apt time, the researchers are motivated to modify Bi_5_Ti_4_O_15_ (n = 4) ceramics using different dopant elements at the A-site (Bi-site) or B-site (Ti-site) or A/B-site simultaneously to achieve the excellent multiferroic and relaxor ferroelectric characters^14,15^. Among the reports on Bi_5_Ti_4_O_15_ (n = 4) layered Aurivillius ceramics, a new series of ferroelectric materials i.e., ABi_4_Ti_4_O_15_ (ABTO) series (A = Ba, Sr, Ca) have been established by Subbarao et al., Kennedy et al., Tellier et al., Rout et al.^2,17–19^. It was observed that an excellent piezoelectric constant with a high ferroelectric phase transition temperature, low processing rate, and lead-free nature of these systems.

In ABTO series, a special attention has been paid to the BaBi_4_Ti_4_O_15_ (BBTO) ceramics (internal structure formulae of (Bi_2_O_2_)^2+^ and (BaBi_2_Ti_4_O_13_)^2−^) because of relaxor ferroelectricity rather than for normal ferroelectric nature in SrBi_4_Ti_4_O_15_ and CaBi_4_Ti_4_O_15_ ceramics^19,20^. Furthermore, BBTO Aurivillius ceramics exhibits the decent piezoelectricity with a high Curie temperature near T_m_ = 410 °C and excellent optical properties^12,21–23^. The BBTO Aurivillius ceramics provides an orthorhombic crystal system with A2_1_am space group at room temperature (RT) and transforms to the tetragonal space group I4/mmm above the transition temperature (T_m_ = 410 °C). Despite these interesting properties, there are few drawbacks in BBTO Aurivillius ceramics, such as the dominance of oxygen vacancies during the high temperature sintering process because of volatilization of bismuth to maintain charge neutrality. In bismuth based Aurivillius ceramics, oxygen vacancies might be preferably present in the vicinity of Bi-ions, which could be the key responsible for ferroelectric character. The dominant oxygen vacancies in bismuth based Aurivillius ferroelectrics provide the several vital negative effects such as fatigue ferroelectric character, pinning at domain walls, enhancing leakage current, trapping charge carriers in defect domains, screening of electric field near the space charge region and impeding the displacement of Ti^4+^ ion^24–26^. To minimize all these drawbacks associated with oxygen vacancies and to improve the piezoelectric/ferroelectric, dielectric character of the ceramics, several attempts have been made using the dopant in A and/or B-site of BBTO ceramics^26–30^. Especially for memories, the low conductivity and low dielectric loss with low fatigue ferroelectric character are the key essentials, and these are introduced by controlling oxygen vacancies in bismuth layered Aurivillius ferroelectrics (BBTO).

In this context, the partial substitution of bismuth by stable trivalent rare-earth (RE) cations has been found to be an effective way to suppress the concentration of oxygen vacancy and improve electrical responses^26–28^. Among the reports, the Sm^3+^/Nd^3+^ ion-substituted bismuth based layered perovskites are often noticed due to enhancement of dielectric constant, ferroelectricity and increment of the fatigue resistance^29,30^. Furthermore, the few researchers reported that structural distortion and diffuse relaxor activity in RE (La^3+^, Sm^3+^, Nd^3+^) ion-substituted BBTO ceramics, which may control deficiency of oxygen vacancies. The A-site substitution by RE^3+^ ion in BBTO Aurivillius oxides provides a structural distortion due to allowing antiphase or in-phase octahedral rotations around the *c* axis that could lead to form polar character^16,29–33^. Moreover, the structural distortion correlated with the ionic radii of A-site cation substitution in BBTO ceramics could affect the lattice parameters as well as the ferroelectric to paraelectric transition temperature (T_m_).

Presently, Khokhar et al. reported the enhancement in ferroelectric properties for La^3+^ ion-substituted BBTO ceramics (20% substitution of La^3+^ ion)^34^. Prakash et al. reported that there is an increment of phase transition temperature (T_m_) whereas the decrease in dielectric loss of Sm^3+^ ion-substituted BBTO ceramics at Ba-site^27^. Furthermore, it is noticed an improved ferroelectric nature with only 5% substitution of Sm^3+^ ion in BBTO ceramics and then decrement in remnant polarization (P_r_) value with increase of substituent. Very recently, 10% Pr^3+^ion-substituted BBTO samples showed an excellent temperature stability, good energy storage density and high efficiency^35^. In view of the above, the subtle substitution of RE^3+^ ion in BBTO Aurivillius ceramics can be suitable for the future high temperature piezoelectric device applications. Therefore, it has been selected the first four rare-earth (RE^3+^) ions in periodic table within very specified substitution in BBTO ceramics. The general formula can be represented as BaBi_4−x_RE_x_Ti_4_O_15_, (*x* = 0.1, RE = La, Pr, Nd, and Sm) within the identified limit. In this paper, we have investigated the structural parameters using Rietveld refinement technique, ferroelectric to paraelectric phase transition, diffuse relaxor nature along with temperature dependent dielectric study and conductivity studies in wide temperature and frequency intervals. Furthermore, polarization vs. electric field (P–E) hysteresis loops at RT are investigated to determine the role of energy density in present compound.

## Experimental details

The RE-substituted BaBi_4−x_RE_x_Ti_4_O_15_ (x = 0.10, RE = La, Pr, Nd, and Sm) 4-layered Aurivillius ceramic oxides were synthesized through conventional solid-state reaction method using high-purity of Bi_2_O_3_ (99.99%), TiO_2_ (99%), BaCO_3_ (99%), and RE_2_O_3_ (La_2_O_3_; Pr_2_O_3_; Nd_2_O_3_; and Sm_2_O_3_) powders. The stoichiometric amounts of precursor powders were mixed thoroughly in an acetone medium using agate mortar to obtain a homogeneous mixture. An extra 5 wt% of Bi_2_O_3_ powder was added to the mixture to compensate the loss in bismuth oxides at elevated temperatures. Repeated grinding and calcinations were carried out at 800 °C, and 900 °C for 6 h, respectively. The calcined powder was reground using 5 wt% of polyvinyl alcohol (PVA) as a binder before being pressed bilaterally into pellets. Subsequently, the pressed pellets were sintered at 1050 °C for 6 h to obtain a dense BaBi_4−x_RE_x_Ti_4_O_15_ ceramics. The phase purity and crystallinity of the ceramics were investigated using powder X-ray diffraction technique for CuK_α_-radiation of an X-ray diffractometer (PHILIPS-PW3373 XPERT-PRO) over the angular range 20° ≤ 2θ ≤ 80°. The grain growth and surface morphology of the sintered pellets were investigated using field-emission scanning electron microscopy (FE-SEM, Sirion 200, FEI Company). The silver (Ag) electrodes were coated on the sintered pellets for electrical measurements. Ferroelectric properties were studied by P-E loop (polarization (*P*) vs. electric field (*E*)) measurement system as performed using a TF-Analyzer 2000 (aix ACCT systems, GmbH) on the silver-coated pellets. Temperature dependent dielectric studies and conductivity analyses were performed with a Wayne Kerr 6500B impedance analyzer in a broad range of temperatures from 30 to 500 °C and frequencies of 10 Hz to 1 MHz.

## Results and discussion

### Structural phase transitions

**Figure 1:**
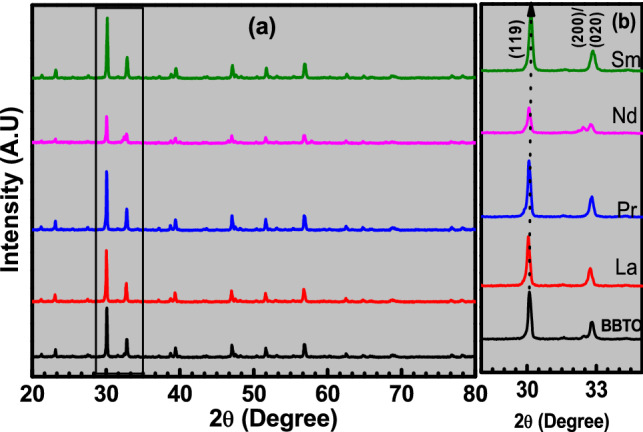
(**a**) Room temperature XRD patterns of pure BaBi_4_Ti_4_O_15_ and RE-substituted BaBi_4−x_RE_x_Ti_4_O_15_ (x = 0.10, RE = La, Pr, Nd, and Sm) ceramics, respectively, and (b) the magnified view of intense peak in the region of 30° ≤ 2θ ≤ 33°.

The XRD patterns of RE-substituted BaBi_4−x_RE_x_Ti_4_O_15_ (“x = 0 and 0.10”, RE = La, Pr, Nd, and Sm) (denoted as BBTO-pc for pristine compound, BBTO-La, BBTO-Pr, BBTO-Nd, and BBTO-Sm) ceramics are measured at RT as shown in Fig. 1a. The observed diffraction peaks are well matched with standard diffraction patterns (ICSD-99501) of BaBi_4_Ti_4_O_15_ Aurivillius phase with A2_1_am space group. The diffraction peaks (119) and (200), as presented in Fig. 1b shift towards the higher angle indicating a reduction in *d*-spacing. This suggests a decrease in the unit cell volume with substitution of RE^3+^ ion in BBTO ceramics. The structure of Aurivillius ceramics was analysed by the Rietveld refinement technique using FullProf software version 2019, with adoption of pseudo-Voigt profile function. It is well reported at RT that the structure of BBTO ceramics has been argued to possess a stable orthorhombic crystal system with A2_1_am space group^27,34,36^. The refinement for BBTO-pc has been initiated by adopting an orthorhombic unit cell with A2_1_am space group, but without deducing the backing signal. In the refinement process, background was modelled using a linear interpolation with refinable heights. The parameter corrections were also considered during the refinement process^37^. Nevertheless, experimental patterns are in well agreement with calculated XRD pattern reasonably based on the low values of refined reliability factors, which are listed in Table 1. It has been found that the refined structure of the BBTO-pc is an orthorhombic crystal system with A2_1_am space group. In addition to that, the detail refined parameters are reported in Table 2 for BBTO and RE-substituted BBTO ceramics.

**Table 1:** Physical parameters of pure BaBi_4_Ti_4_O_15_ and RE-substituted BaBi_4−x_RE_x_Ti_4_O_15_ (x = 0.10, RE = La, Pr, Nd, and Sm) ceramics.

Sample	Ionic radar (Å)	*a *(Å)	*b *(Å)	*c *(Å)	*V *(Å)^3^	χ^2^	(R_B_, R_F_)
BBTO PC	Bi (1.38)	5.46990 ± 0.00033	5.45980 ± 0.00032	41.87000 ± 0.00025	1249.55	3.17	(9.12, 8.23)
BBTO x = La	La (1.36)	5.46970 ± 0.00037	5.45910 ± 0.00033	41.84600 ± 0.00025	1249.49	3.03	(8.14, 10.10)
BBTO x = Pr	Pr (1.32)	5.46950 ± 0.00035	5.45790 ± 0.00037	41.83900 ± 0.00027	1248.88	2.91	(9.26, 8.97)
BBTO x = Nd	Nd (1.29)	5.46930 ± 0.00042	5.45730 ± 0.00039	41.82000 ± 0.00024	1248.30	3.48	(8.96, 10.09)
BBTO x = Sm	Sm (1.26)	5.46910 ± 0.00038	5.45630 ± 0.00035	41.81500 ± 0.00028	1247.80	2.87	(8.18, 8.74)

**Figure 2:**
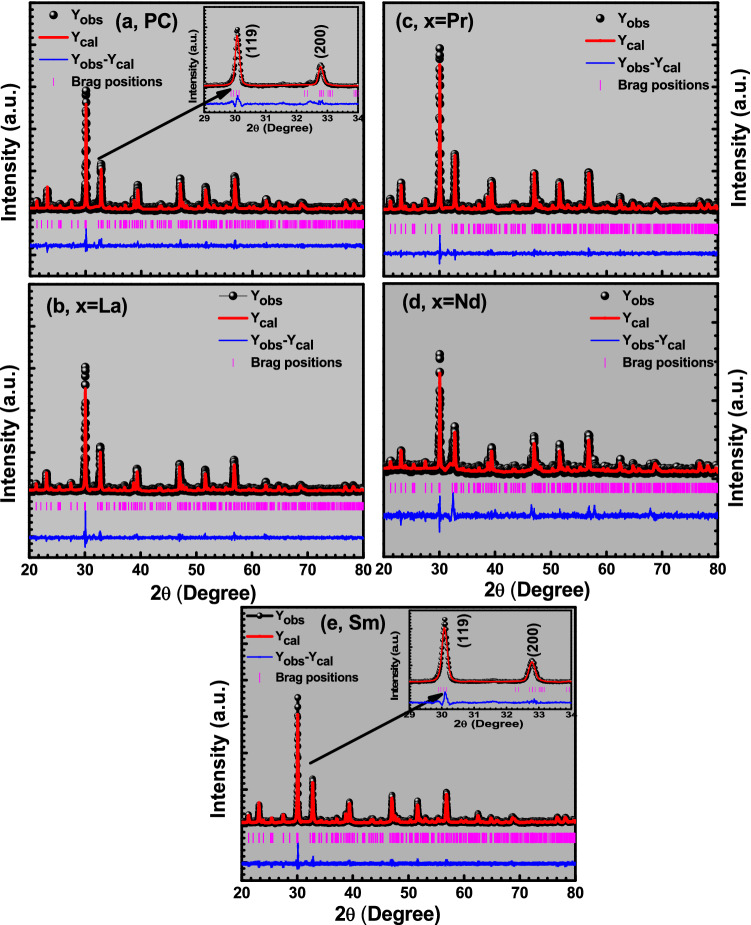
(**a**–**e**) Rietveld refinements of XRD patterns of pure BaBi_4_Ti_4_O_15_ and RE-substituted BaBi_4−x_RE_x_Ti_4_O_15_ (x = 0.10, RE = La, Pr, Nd, and Sm) Aurivillius ceramics. Inset of (**a**) shows the magnified view of (119) and (200) diffraction peaks.

The RE-substituted BBTO ceramics were refined with the aid of BBTO refined lattice parameters, scaling factor and shape parameters. Here, only 0.10 atomic occupation of Bi^3+^ ion was replaced with RE^3+^-ion to maintain constant sum of the occupancy equal to 4 at bismuth site. The experimental pattern is well matched with the calculated XRD pattern for RE^3+^ ion-substituted BBTO Aurivillius ceramics through confirmation of an orthorhombic crystal system with A2_1_am space group. Rietveld refined plots of pure BBTO and RE-substituted BBTO ceramics are shown in Fig. 2a–e, and the zoomed pattern of particular (119)_pc_, (200)_pc_ peaks are represented in the inset of Fig. 2a,e. The various structural parameters obtained from refinement are listed in Table 1.

**Table 2:** Rietveld refined parameters of pure BaBi_4_Ti_4_O_15_ and RE-substituted BaBi_4−x_RE_x_Ti_4_O_15_ (x = 0.10, RE = La, Pr, Nd, and Sm) ceramics.

BBTO-parent compound (PC)						RE doped-BBTO (RE = La, Pr, Nd, and Sm)					
Atom	Type	x	y	z	Occupancy	Atom	Type	x	y	z	Occupancy
Bi1	Bi	0.25	0.25	0.221	0.895	Bi1	Bi	0.25	0.25	0.221	0.895
Bi1	Bi	0.285	0.25	0	0.435	Bi2	Bi	0.285	0.25	0	0.434
Bi3	Bi	0.267	0.25	0.893	0.65	Bi3	Bi	0.267	0.25	0.893	0.65
Ba1	Ba	0.248	0.25	0.207	0.155	Ba1	Ba	0.248	0.25	0.207	0.105
Ba2	Ba	0.19	0.25	0	0.07	Ba2	Ba	0.19	0.25	0	0.06
Ba3	Ba	0.219	0.25	0.9	0.308	Ba3	Ba	0.219	0.25	0.9	0.398
Ti1	Ti	0.245	0.25	0.55	1	RE	RE	0.248	0.25	0.207	0.022
Ti2	Ti	0.243	0.25	0.346	1	Ti1	Ti	0.245	0.25	0.55	1
O1	O	0.711	0.215	0	0.5	Ti2	Ti	0.243	0.25	0.346	1
O2	O	0.509	0.469	0.548	1	O1	O	0.711	0.215	0	0.5
O3	O	0.446	− 0.033	0.544	1	O2	O	0.509	0.469	0.548	1
O4	O	0.999	0.5	0.25	1	O3	O	0.446	− 0.033	0.544	1
O5	O	0.223	0.242	0.405	1	O4	O	0.999	0.5	0.25	1
O6	O	0.489	0.5	0.356	1	O5	O	0.223	0.242	0.405	1
O7	O	0.489	0	0.356	1	O6	O	0.489	0.5	0.356	1
O8	O	0.246	0.25	0.695	1	O7	O	0.489	0	0.356	1
						O8	O	0.246	0.25	0.695	1

The observed slight decrement in lattice constant and unit cell volume for RE^3+^ ion substitution in BBTO ceramics are listed in Table 1. The internal structure of BBTO Aurivillius layered perovskite ceramics is associated with two regular stacking of fluorite-like [Bi_2_O_2_]^2+^ slabs and perovskite-like [BaBi_2_Ti_4_O_13_]^2−^ blocks. Here, the internal Bi^3+^ ion has contained in two sites, one at [Bi_2_O_2_]^2+^ layers where Bi_2_ have 8 coordination number (CN), other at A-site for [BaBi_2_Ti_4_O_13_]^2−^ blocks where BaBi_2_ adopts 12 CN. For the abovementioned reasons, the internal stacking of [Bi_2_O_2_]^2+^ layers and perovskite unit cell [BaBi_2_Ti_4_O_13_]^2−^ blocks are always remained under the compressive and tensile stresses. The stresses arise due to a mismatch in ionic radii of RE^3+^ ions and A-site (Ba^2+^/Bi^3+^) ions. Thus, this could release the stress between perovskite unit cells and [Bi_2_O_2_]^2+^ layers. As a result, the structural distortion will be decreased in substituted Aurivillius structures^38,39^. Henceforth, the decrease in lattice parameters as well as in unit cell volume for RE^3+^ ion substitution in BBTO ceramics was noticed.

Furthermore, it is correlated in terms of tolerance factor ‘*t*’ given by ionic radii of A- and B-site cations associated to TiO_6_ octahedra and AO planes in the Aurivillius perovskite block. Here, the tolerance factor ‘*t*’ can be defined as^38,39^

1 $$t=\frac{{R}_{A}+{R}_{O}}{\sqrt{2} ({R}_{B}+{R}_{O})},$$

where R_A_, R_B_ and R_O_ are the effective ionic radii for the A-, B-site cations and oxygen anion in ABO_3_ perovskite, respectively. Generally, a lower ‘*t*’ value suggests a higher degree of structural distortion, which depends upon the ionic radii of A and B-site cations in Aurivillius perovskite [A_n−1_B_n_O_3n+1_]^2-^ blocks. Here, the ionic radii of A-, B-site cations and oxygen anion can be expressed more clearly as, $R_{A} = R_{{Bi^{{3 + }} (IIX)}} = 1.38\mathop {\text{A}}\limits^{ \circ }$, ${R}_{A}={R}_{{Ba}^{2+}(IIX)}=1.61 \mathop {\text{A}}\limits^{ \circ }$ ${R}_{B}= {R}_{{Ti}^{4+}(IV)}=0.605 \mathop {\text{A}}\limits^{ \circ }$, ${R}_{{O}^{2-}}=1.40 \mathop {\text{A}}\limits^{ \circ }$, respectively. The ionic radii of B-site ions and oxygen ion remains unaltered in the RE-substituted BBTO layered ceramics. In the interim, the occupied RE^3+^ ions have 12 CN in perovskites for the ionic radii of La^3+^ = 1.360 Å to Sm^3+^ = 1.26 Å, which are smaller than that of Bi^3+^ ion. This could lead to a slight decrease in tolerance values in RE-substituted BBTO in comparison to pristine one^40,41^. Henceforth, a decrease in ‘*t*’ value indicates the distortion in BO_6_ octahedra caused by decrease in average ionic radii of A-site cations. Furthermore, the reduction of octahedral size may distort the lattice in *ab*-plane, henceforth the shrinkage of lattice distortion for all the axes could lead to decrease in lattice constant and unit cell volume.

**Figure 3:**
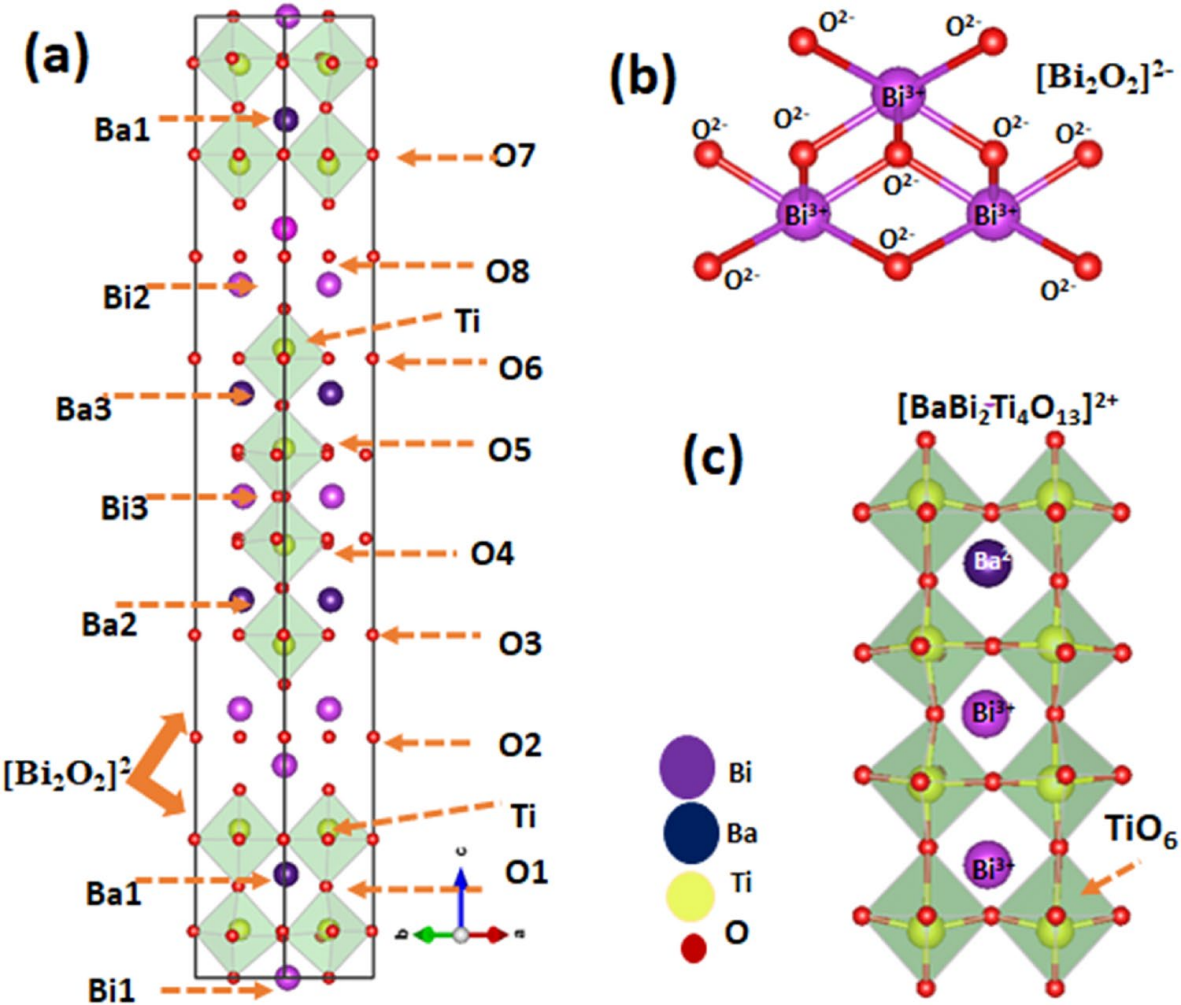
Schematic crystal structural diagram performed through the Vesta software by using the output of Rietveld refinement crystallographic information file (cif). (**a**) Crystal structure of BaBi_3.90_Sm_0.1_Ti_4_O_15_ unit cell with A2_1_am space group along *c*-axis. (**b**,**c**) Representation of the (Bi_2_O_2_)^2+^ fluorite like layered units with geometrically alternating arrangement of 4-layered (BaBi_2_Ti_4_O_13_)^2−^ perovskites in connection to TiO_6_ centred octahedra.

Figure 3a–c exhibits the schematic structural view along *c*-axis [001] of the Sm^3+^ ion-substituted BBTO unit cell. These were drawn using the structural parameters obtained through Rietveld refinement program. The sandwich geometry for alternate arrangement of (BaBi_2_Ti_4_O_13_)^2−^ and fluorite-like layers of (Bi_2_O_2_)^2+^ ions along with oxygen atomic positions are represented within the unit cell^39,42^. Figure 3b,c represents the (Bi_2_O_2_)^2+^ layered units with disordered cations in the A-site and TiO_6_ centred octahedra cross-linked array in four-layered perovskite for BaBi_4_Ti_4_O_15_ unit cell. This undergoes an obvious tilting due to the distortion of excessive asymmetric coordination of Bi^3+^/Ba^2+^/Sm^3+^ ions^27,39^. The details of Wyckoff positions (*x, y, z*) of the relevant atomic sites, as well as bond lengths, and bond angles for BO_6_ octahedra in pristine and RE^3+^ ion substituted BBTO ceramics are listed in Tables 2 and 3, respectively.

**Table 3:** Few Selected bond distances and bond angles in BO_6_ octahedra (Ti–O–Ti) for all BBTO ceramics.

BBTO-PC	BBTO-La	BBTO-Pr	BBTO-Nd	BBTO-Sm
**Bond distance (Å)**				
(Ti1–O1) = 1.9158	(Ti1–O1) = 1.8770	(O1–Ti1) = 2.10875	(O1–Ti1) = 2.1078	(O1–Ti1) = 2.10703
(O2–Ti1) = 2.0048	(Ti1–O2) = 2.0056	(O2–Ti1) = 1.87524	(O2–Ti1) = 1.8766	(O2–Ti1) = 2.00474
(O3–Ti1) = 2.0359	(Ti1–O3) = 2.0369	(O3–Ti1) = 2.03502	(O3–Ti1) = 2.0364	(O3–Ti1) = 1.91548
(Ti1–O4) = 1.8829	(Ti1–O4) = 1.8814	(Ti1–O4) = 2.03502	(O4–Ti1) = 2.0051	(O4–Ti1) = 2.00474
(Ti1–O5) = 1.9816	(Ti2–O5) = 1.9617	O5–Ti1) = 1.88285	(O5–Ti1) = 1.8820	(O5–Ti1) = 1.88129
(Ti1–O6) = 2.0023	(Ti2–O6) = 1.9949	(O6–Ti1) = 1.91450	(O6–Ti1) = 2.1078	(O6–Ti1) = 2.03625
**Bond angle (**$^\circ$**)**				
Ti1–O1–Ti1 = 165.55	Ti1–O1–Ti1 = 168.21	Ti1–O1–Ti1 = 168.53	(Ti1–O1–Ti1) = 171.17	(Ti1–O1–Ti1) = 171.22
Ti1–O3–Ti1 = 158.837	Ti1–O3–Ti1 = 155.63	Ti1–O3–Ti1 = 156.84	(Ti1–O3–Ti1) = 159.48	(Ti1–O3–Ti1) = 161.48
O2–Ti1–O3 = 169.493	O2–Ti1–O3 = 169.50	O2–Ti1–O3 = 168.818	(O2–Ti2–O3) = 165.17	(O2–Ti2–O3) = 168.21
O6–Ti2–O7 = 158.130	O6–Ti2–O7 = 155.18	O6–Ti2–O7 = 155.160	(O7–Ti1–O6) = 158.19	(O7–Ti1–O6) = 161.29
Ti2–O6–Ti2 = 156.121	Ti2–O6–Ti2 = 158.12	Ti2–O6–Ti2 = 158.139	(Ti2–O6–Ti2) = 159.23	(Ti2–O6–Ti2) = 160.12
Titling angle	Titling angle	Titling angle	Titling angle	Titling angle
(O2–O2–O3) = 10.345	(O2–O2–O3) = 8.46	(O2–O2–O3) = 8.282	(O2–O2–O3) = 7.451	O2–O2–O3 = 7.1804

A strong correlation between structural distortion, breaking of inversion symmetry, and internal ferroelectric phase transitions can be established with the help of internal rearrangement of BO_6_ octahedra before and after substitution of RE^3+^ ions. For the atomic displacement within the BO_6_ octahedra due to the substitution of the RE element at the Bi-site, the selected bond angles and bond distances are summarized in Table 3. For the core atomic displacement of Ti(1)–O in BO_6_ octahedra along the three axes, the changes in Ti–O–Ti bond angle and the average Ti–O distance occur for all RE-substituted BBTO ceramics compared to pristine one. These results are also consistent with the inclusion of RE^3+^ ion at the *A*(1)-site in the perovskite layer, thus the changes in bond distances are related to the ionic radii of the RE^3+^ ions. Additionally, there is also a slight reduction of the rotation angle of *B*O_6_ octahedra around the *c*-axis for RE^3+^ ion substitution as observed in Supplementary Fig. S1a–c. This is evidenced through the reduction of bond angles by less than 180° as listed in Table 4 and also visually depicted in Supplementary Fig. S1d–f. The octahedral tilting in Aurivillius phases with space group *A*21*am* takes place around all the three axes, where it will be out-of-phase tilting along the *a* and *b*-axes or in-phase tilting along the *c*-axis. Therefore, the tilting around the *a*-axis is quantified by the Ti–O(1)–Ti bond angle^9,24^, whereas the tilting in the *ab*-plane is defined by the relative rotation angle between neighboring octahedra around the *c*-axis^24,26^. The *B*O_6_ octahedra are considerably tilted around the *c*-axis by 10.345° in BBTO ceramics. This is detected in Ti(1)–O(1)–Ti(1) bond angle by 165.55° as shown in Supplementary Fig. S1a–c. The RE^3+^ ion substitution causes a substantial decrease in tilt angle with respect to ionic radii of RE^3+^ ion, as detected to be 8.28° and 7.18° for BBTO-Pr and BBTO-Sm, respectively. This implies that a decrease in the degree of tilting reduces the structural distortion leading to a decrease in lattice parameter along the *c*-axis as well as *ab*-plane. Thus, this indicates a decreased orthorhombicity leads to a more symmetric structure for RE^3+^ ion substitutions in BBTO ceramics.

**Table 4:** The temperature dependent exponent (n_2_) and dc conductivity ${\sigma }_{dc}\left(0\right)$ values of pure BaBi_4_Ti_4_O_15_ and RE-substituted BaBi_4−x_RE_x_Ti_4_O_15_ (x = 0.10, RE = La, Pr, Nd, and Sm) ceramics.

Temperature (℃)	σ_dc_ (BBTO-PC)	σ_dc_ (BBTO-La)	σ_dc_ (BBTO-Pr)	σ_dc_ (BBTO-Nd)	σ_dc_ (BBTO-Sm)
200	1.38 $\times$ 10^–8^	7.75 $\times$ 10^–8^	7.73 $\times$ 10^–8^	5.08 $\times$ 10^–8^	4.60 $\times$ 10^–9^
240	2.7 $\times$ 10^–8^	1.68 $\times$ 10^–7^	1.5 $0 \times$ 10^–7^	1.40 $\times$ 10^–7^	3.38 $\times$ 10^–9^
280	1.06 $\times$ 10^–8^	3.30 $\times$ 10^–7^	2.95 $\times$ 10^–7^	3.23 $\times$ 10^–7^	5.58 $\times$ 10^–8^
300	1.50 $\times$ 10^–7^	5.01 $\times$ 10^–7^	4.00 $\times$ 10^–7^	4.35 $\times$ 10^–7^	1.60 $\times$ 10^–7^
340	3.22 $\times$ 10^–7^	6.60 $\times$ 10^–7^	9.56 $\times$ 10^–7^	1.17 $\times$ 10^–6^	6.92 $\times$ 10^–7^
380	1.25 $\times$ 10^–6^	1.6 $0 \times$ 10^–6^	2.27 $\times$ 10^–6^	3.13 $\times$ 10^–6^	1.50 $\times$ 10^–6^
400	3.23 $\times$ 10^–6^	2.63 $\times$ 10^–6^	3.00 $\times$ 10^–6^	4.30 $\times$ 10^–6^	3.20 $\times$ 10^–6^
420	4.05 $\times$ 10^–6^	2.73 $\times$ 10^–6^	3.48 $\times$ 10^–6^	5.2 $0 \times$ 10^–6^	5.50 $\times$ 10^–6^
440	4.02 $\times$ 10^–6^	2.87 $\times$ 10^–6^	4.30 $\times$ 10^–6^	6.36 $\times$ 10^–6^	8.78 $\times$ 10^–6^
	0.86 ≤ n_2_ ≤ 0.79	0.85 ≤ n_2_ ≤ 0.99	0.88 ≤ n_2_ ≤ 1.03	0.84 ≤ n_2_ ≤ 1.04	0.86 ≤ n_2_ ≤ 1.01

**Figure 4:**
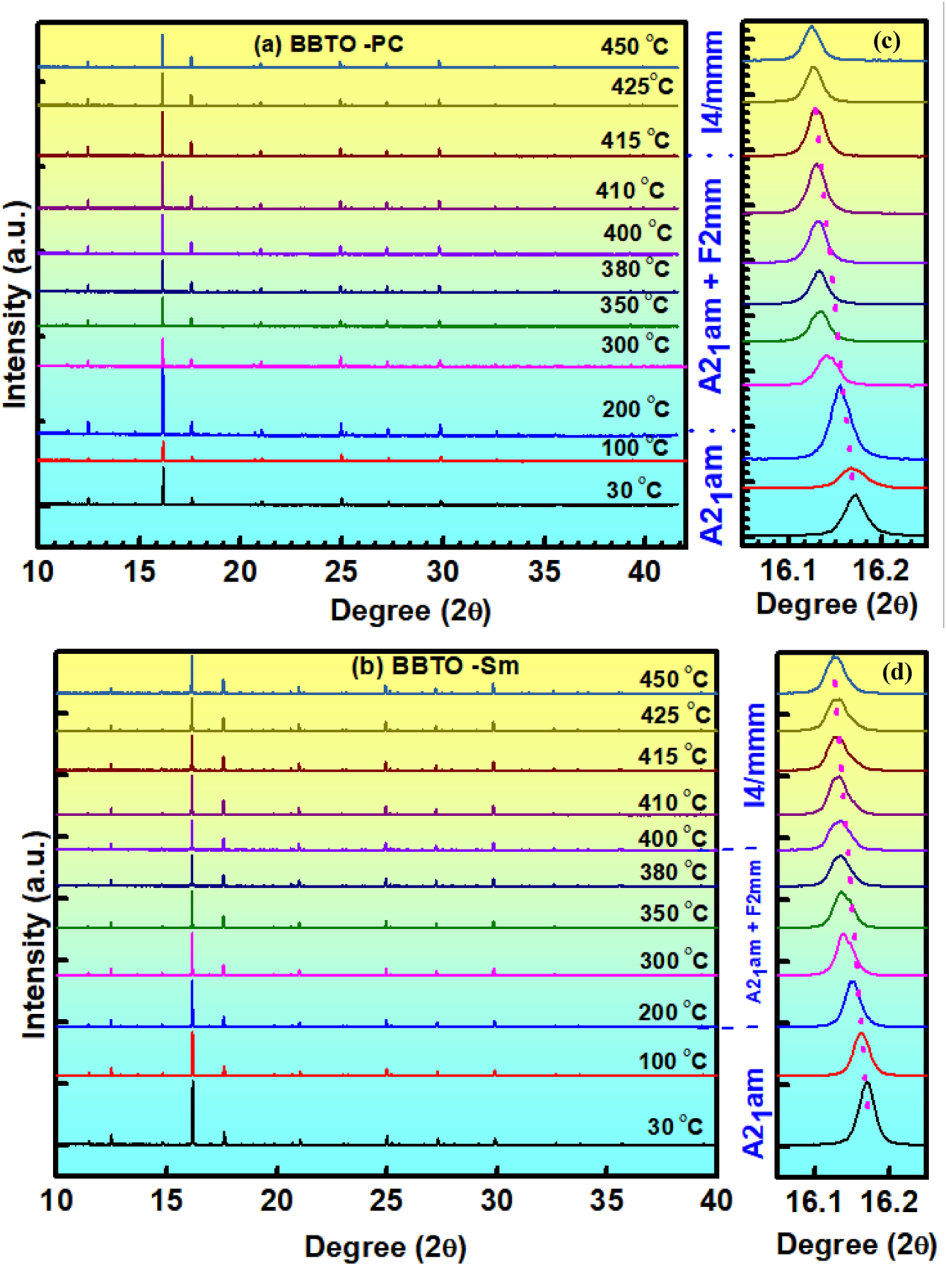
(**a**–**d**) Temperature dependent synchrotron XRD patterns of pure BBTO and Sm-BBTO ceramics for the potential strength of 14.7 keV.

Although BBTO is known to be a good ferroelectric having relaxor response, but the clear understanding about relaxor behaviour of BBTO and RE^3+^ ion substituted BBTO ceramics with structure-dependent phase transition is still not so much attained. Hence, we carried out the temperature-dependent synchrotron XRD of all the Aurivillius ceramics performed from 25 to 450 °C. The pure BBTO exhibits orthorhombic (A2_1_am) structure at RT, which undergoes a gradual transformation into tetragonal (I4/mmm) structure at ~ 415 °C. Figure 4a,b exhibits the temperature dependent synchrotron XRD pattern with a clear peak shift at 350 °C. A clear peak shift is noticed for the Bragg angle from 16° to 16.2° at 200 °C, which is started to diverge completely at 415 °C. This resembles a clear structural phase modification towards a sub-space group of *A*2_1_*am* symmetry at 200 °C onwards in BBTO ceramics. The orthorhombic system with *A*2_1_*am* space group is associated to three more sub-group symmetries with non-polar structure: (1) F2mm, (2) Amam, and (3) Abam. At the specific temperature, the redistribution of cations or reorientation of atomic sites leads to form the sub-group symmetry of *A*21*am* space group. The most probable non-polar structure within *A*2_1_*am* space group with lower sub-group symmetry of F2mm is experimentally detected. Therefore, on a weighted average over all possible configurations and corresponding polar structure lead to support the formation of a high symmetry *I*4*/mmm* space group at 415 °C. It means there is a coexistence of dual phase (orthorhombic-A2_1_am + Orthorhombic-F2mm) at the temperature from 200 to 415 °C, and there is a compete transformation of polar tetragonal (I4/mmm) structure at ~ 415 °C. In addition to that, the Sm^3+^ ion-substituted BBTO ceramics also exhibit the orthorhombic (A2_1_am) phase at RT along with dual phase (orthorhombic-A2_1_am + Orthorhombic-F2mm) in the region of 200–380 °C as displayed in Fig. 4c,d. Other than the above, there is a complete phase transformation to tetragonal (I4/mmm) structure at ~ 380 °C. This observation led to solve the mystery behind the appearance of relaxor behaviour and its effect on diffuse phase transition in dielectric study for the single structural phase transformation into a coexistence of orthorhombic and tetragonal phases as discussed in the next part.

### Surface morphology

**Figure 5:**
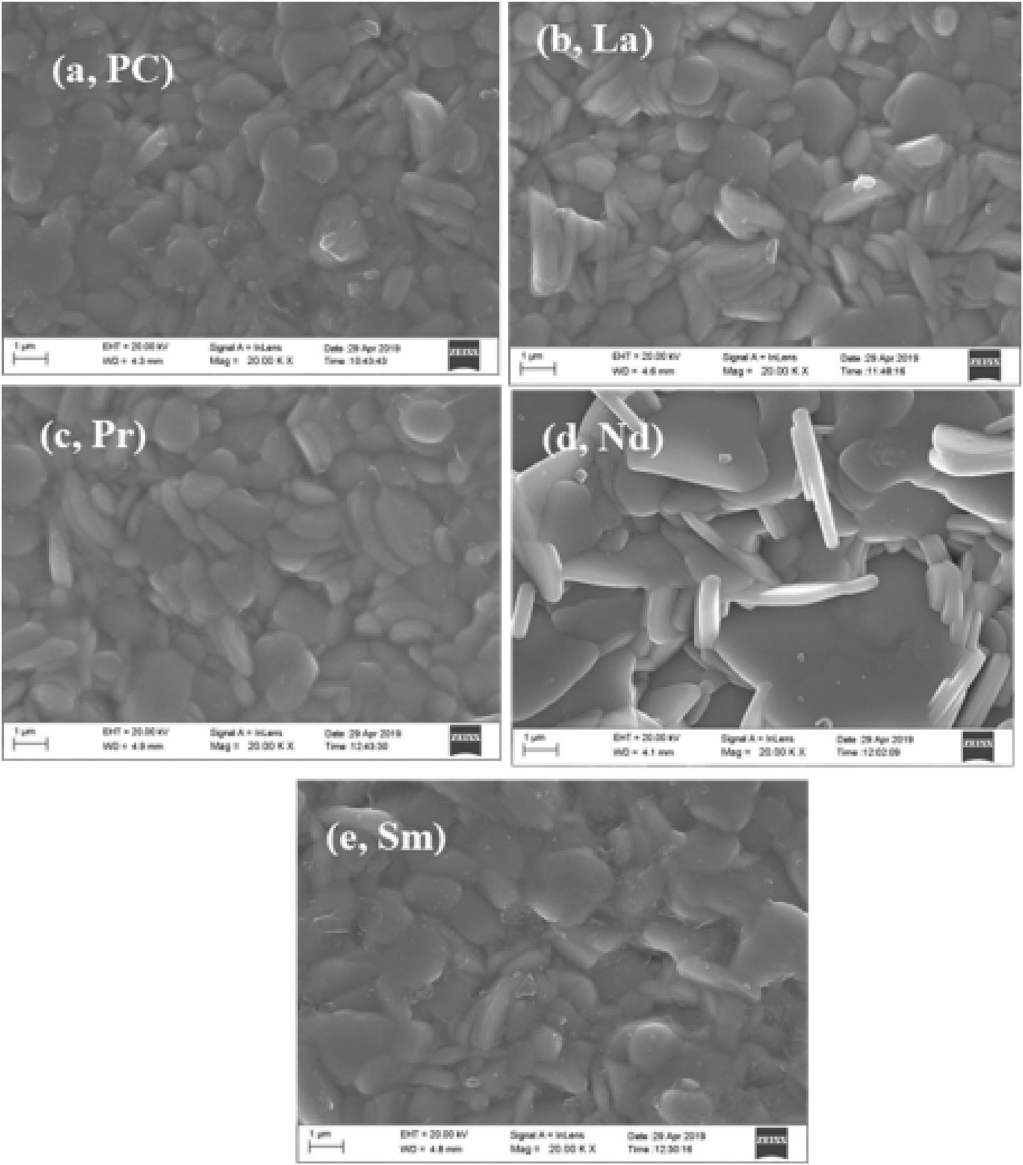
Typical FESEM images for a cross-sectional surface of BaBi_4_Ti_4_O_15_ and RE-substituted BaBi_4−x_RE_x_Ti_4_O_15_ (x = 0.10, RE = La, Pr, Nd, and Sm) ceramics: (**a**) pure $\mathrm{BBTO}$, (**b**) BBTO-La, (**c**) BBTO-Pr, (**d**) BBTO-Nd, and (**e**) BBTO-Sm ceramics, respectively.

**Figure 6:**
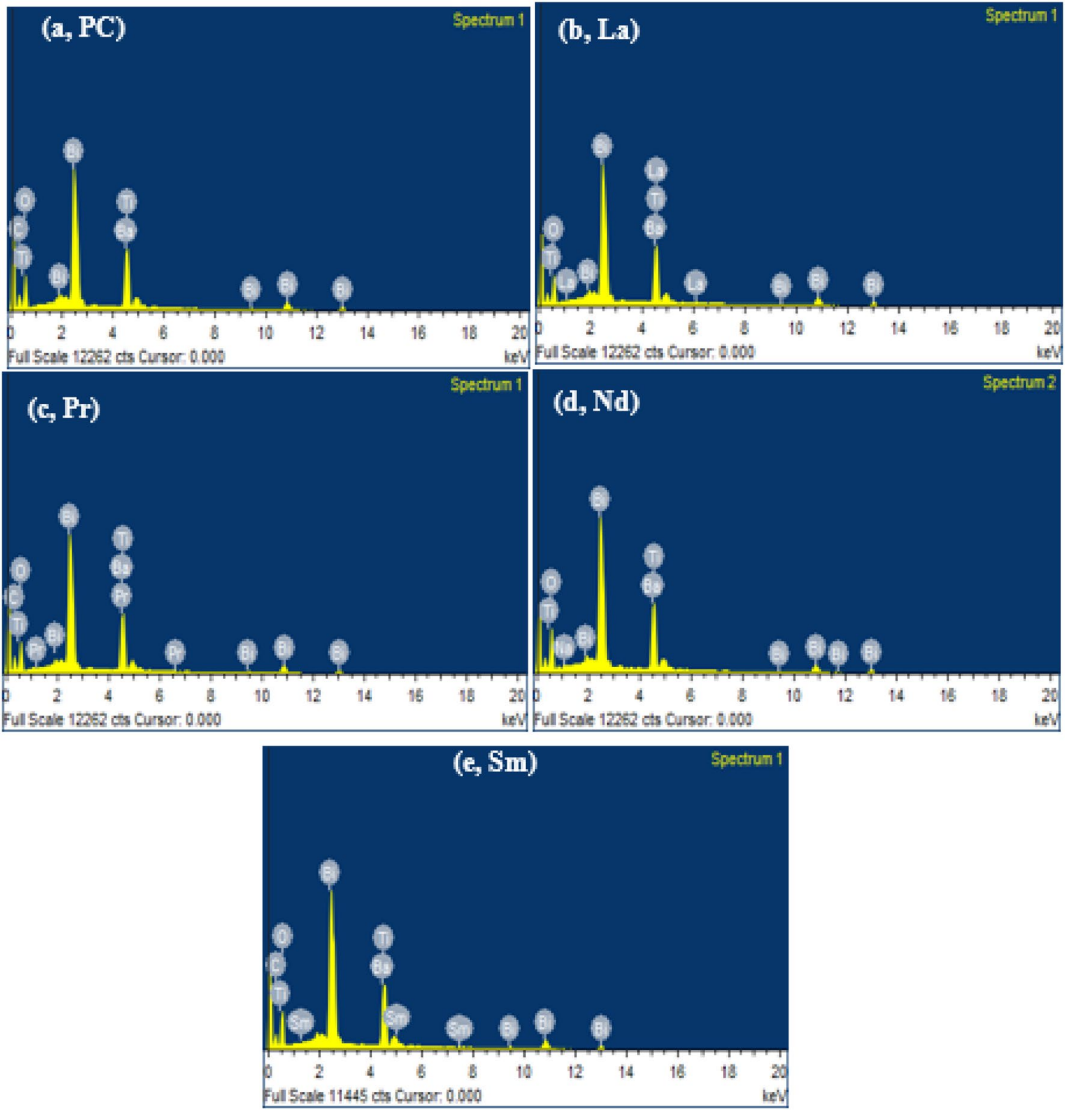
Elemental dispersive spectrum (EDX) of BaBi_4_Ti_4_O_15_ and RE-substituted BaBi_4−x_RE_x_Ti_4_O_15_ (x = 0.10, RE = La, Pr, Nd, and Sm): (**a**) pure $\mathrm{BBTO}$ (**b**) BBTO-La, (**c**) BBTO-Pr, (**d**) BBTO-Nd, and (**e**) BBTO-Sm ceramics, respectively.

A well-polished pellet was used to study the cross-sectional surface morphology, grain growth mechanism and elemental analysis as performed through scanning electron microscopy (SEM). The SEM images of the RE-substituted BBTO samples were depicted in Fig. 5a–e. The observed surface morphology illustrates the randomly oriented plate-like grains that stand for the characteristics of bismuth layered Aurivillius ceramics. The calculated density of the pellets (Table 5) provides to be 7.21, 7.23, 7.15, 7.11, and 7.14 gm/cm^3^ for BBTO-PC, BBTO-La, BBTO-Pr, BBTO-Nd, and BBTO-Sm ceramics, respectively. The provided values are much greater than 90% of the theoretical density. The average grain size is estimated to be 1.14 µm ≤ *d* ≤ 1.58 µm. Obviously, a slight decrease in grain size with the insertion of increasing RE^3+^ ionic radii was observed. The slight decrease in grain size with RE^3+^ ions substitution could be the rate of less diffusivity during the sintering process. In contrast, the Nd^3+^ ion-substituted BBTO samples display thin sharp plate-like grains. This specified the oriented grain growth significantly higher in the direction perpendicular to *c*-axis. Therefore, a significant length of the plate-like grain is much bigger than the thickness as noted in Nd^3+^ ion-substituted BBTO ceramics^24,30^. To confirm the chemical compositions and compositional homogeneity, a spot EDS or EDX (Elemental dispersive spectrum) was carried out on different grain surface interiors. A representative EDX spectrum of all the ceramics were shown in Fig. 6a–e. This approves the presence of RE elements as well as uniformity in elemental incorporation in the BBTO system.

**Table 5:** Electrical parameters of the pure BaBi_4_Ti_4_O_15_ and RE^3+^ ion-substituted BaBi_4-x_RE_x_Ti_4_O_15_ (x = 0.10, RE = La, Pr, Nd, and Sm) ceramics.

Electrical parameters	(BBTO-PC)	(BBTO-La)	(BBTO-Pr)	(BBTO-Nd)	(BBTO-Sm)
T_m_ (°C)	415	392	390	388	380
Tolerance (t)	0.915	0.913	0.911	0.900	0.900
ρ (gm/cm^3^)	7.21	7.23	7.15	7.11	7.14
${\varepsilon }_{r}$ at RT @1 MHz	160	239	248	264	175
E_a_ (meV)	5.20	1.52	7.73	15.02	12.05
ΔT _(relax)_	12	15	15	15	15
σ_ac_ (S/cm)	1.38 $\times$ 10^–8^	7.75 $\times$ 10^–8^	7.73 $\times$ 10^–8^	5.08 $\times$ 10^–8^	4.60 $\times$ 10^–9^
γ	1.49	1.46	1.43	1.98	1.78
2P_r_ (μC/cm^2^)	1.74	1.66	0.49	0.94	0.43

### Temperature dependent dielectric spectroscopy

**Figure 7:**
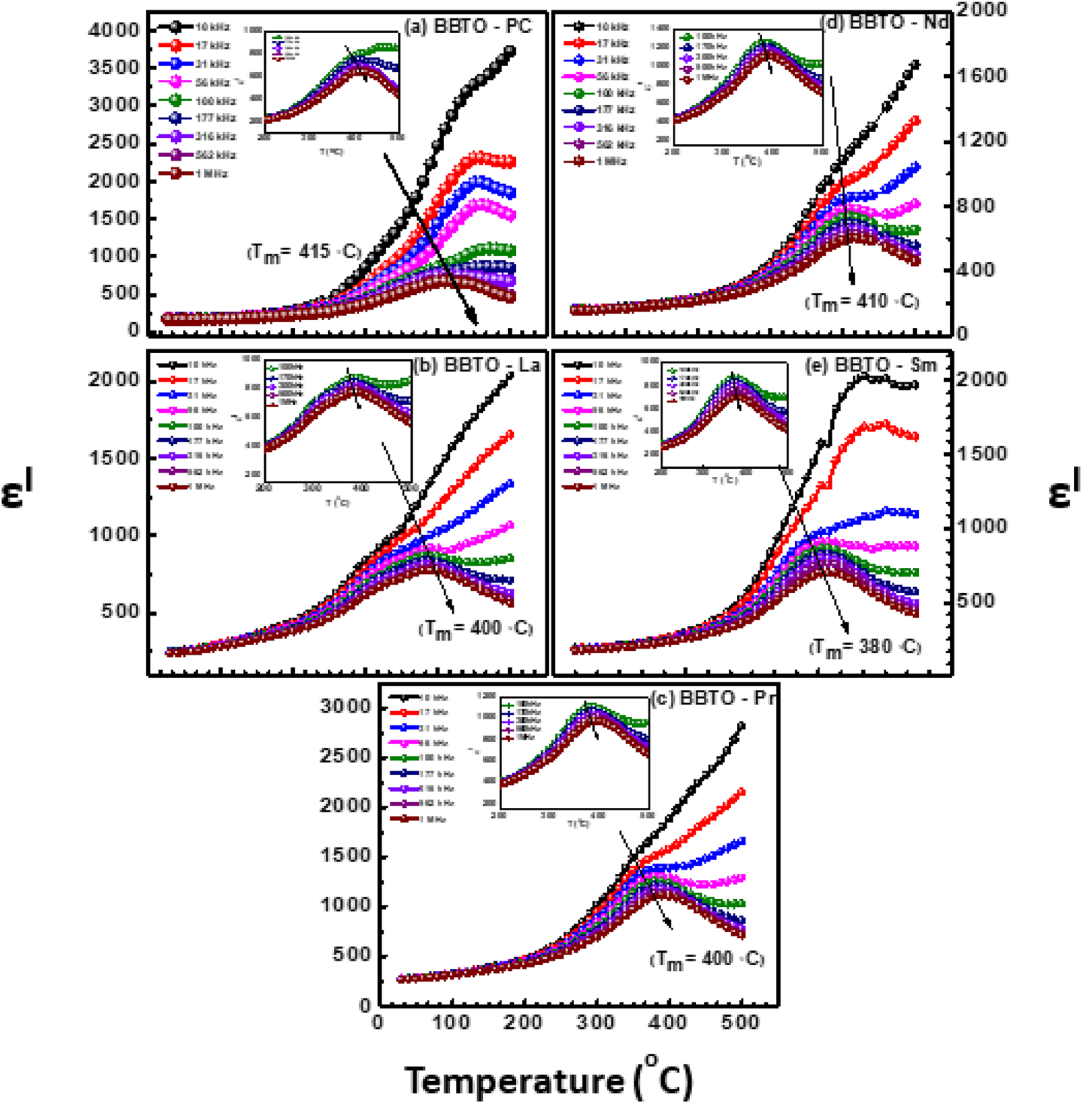
Variation of dielectric constant with temperature at fixed frequency (10 kHz to 1 MHz) intervals of pure BaBi_4_Ti_4_O_15_ and RE-substituted BaBi_4−x_RE_x_Ti_4_O_15_ (x = 0.10, RE = La, Pr, Nd, and Sm) ceramics: (**a**) pure $\mathrm{BBTO},$ (**b**) BBTO-La, (**c**) BBTO-Pr, (**d**) BBTO-Nd, and (**e**) BBTO-Sm. Inset of Figure shows the magnified view near Curie temperature region.

Figure 7a–e illustrates the temperature dependent dielectric constant (${\varepsilon }_{r}$) of pure BBTO and RE-substituted BBTO Aurivillius ceramics in the range from 30 to 500 °C at the selective frequency intervals (10 kHz to 1 MHz). The RT dielectric constant and dielectric loss at 1 MHz are to be 160.65 and 0⋅021, respectively for pure BBTO ceramics. From the available literature, the pure BBTO ceramic sample exhibits a relaxor ferroelectric behaviour at T_m_ ~ 420 °C, which is known as Curie temperature. The signified T_m_ corresponds to crystal structure transformation from orthorhombic to tetragonal (I4/mmm)^21,23,30^. However, in the present investigation, the pure BBTO ceramics exhibit a T_m_ at ~ 415 °C @ 1 MHz frequency. Thus, the T_m_ shifted towards the high temperature region from 396 to 415 °C with an increase of frequency from 50 kHz to 1 MHz as shown in the inset of Fig. 7a. This designates the diffuse relaxor ferroelectric character. The observed temperature and frequency dependent diffuse relaxor dielectric behaviour in BBTO ceramics are in consistent with the previous reports^19–21,36^. Meanwhile, the temperature dependent dielectric loss peaks also exhibit the frequency dependency near 370–400 °C (Supplementary Fig. S2a–e), which are slightly lower than T_m_. Then, above the T_m_, a dramatically increase in dielectric loss is anticipated due to increase in conductivity with temperature. The dielectric loss relaxation phenomenon just below their respective Curie temperature has already been reported in barium based Aurivillius compounds^2,21,43–45^.

A similar feature of RE^3+^ion-substituted BBTO ceramics as illustrated in Fig. 7a–e for frequency and temperature dependent dielectric plots implied a diffuse/relaxor ferroelectric behaviour. The large dielectric dispersion is observed above and below T_m_. A degree of relaxation behaviour calculated with formula ΔT_relax_ = T_50 kHz_ − T_1 MHz_ ≃ 15 °C is found to be constant for all the RE^3+^ion-substituted BBTO ceramics. The dielectric maxima (ε_max_) decrease with increase of frequency as a function of temperature. A slight decrease in T_m_ towards the low temperature region in RE^3+^ion-substituted BBTO ceramics is observed. Furthermore, the RT dielectric constant for pristine, La^3+^, Pr^3+^, Nd^3+^ and Sm^3+^ ion-substituted BBTO ceramics are to be 160, 239, 248, 264, 175, respectively @ frequency of 1 MHz as listed in Table 5. This signifies a decrease in dielectric constant with the increase of RE-atomic number for RE^3+^ ion-substituted BBTO ceramics. In comparison with the pristine BBTO ceramics, the dielectric constant (ε_r_) of RE^3+^ ion-substituted BBTO ceramics is increased. At T_m_, a significant increase in dielectric constant (ε_m_) along with dielectric loss was also observed for RE^3+^ ion substituted BBTO systems. A slight decrease in grain size and increase in thickness of plate like grains with the insertion of increasing RE^3+^ ionic radii exhibit the movement of domain walls, contributing to the increase in magnitude of ε_m_ at T_m_.

To estimate the origin of diffuse/relaxor activity in pure and RE^3+^ ion-substituted BBTO ceramics, the temperature dependent dielectric plots are presented in the inset of Fig. 7b–e at fixed frequencies. The dielectric (ε^l^ (T)) plots exhibit a merging at fixed frequency region (50 kHz to 1 MHz), and a fixed temperature interval (T_m_ ˃ 80 °C). Furthermore, a large dielectric dispersion at low frequency and high temperature region was observed. It signifies the observed relaxor activity was not like that of classical relaxor ferroelectrics. The classical relaxor ferroelectrics show a complete merge of the dielectric spectra above T_m_ over a wide frequency and temperature regime. Therefore, to explain internal dielectric diffusivity and relaxor activity of RE^3+^ ion-substituted BBTO ceramics, both the modified Curie–Weiss law and Vogul–Fulcher (VF) non-linear equations are utilized. Furthermore, the dielectric loss relaxation peaks with its frequency dependency were noted at slightly lower than Curie temperature (T_m_) for all RE^3+^ ion-substituted BBTO ceramics (Supplementary Fig. S2a–e)^46,48^.

To describe the diffuse relaxor activity of ferroelectric phase transition, the modified empirical Curie–Weiss law was proposed by Uchino and Nomura as given in Eq. (2)^30^.

2 $$\frac{1}{{\varepsilon }^{l}}-\frac{1}{{\varepsilon }_{max}^{l}}= \frac{{\left(T-{T}_{m}\right)}^{\gamma }}{C}\left(\mathrm{T }>\mathrm{ Tm}\right).$$

**Figure 8:**
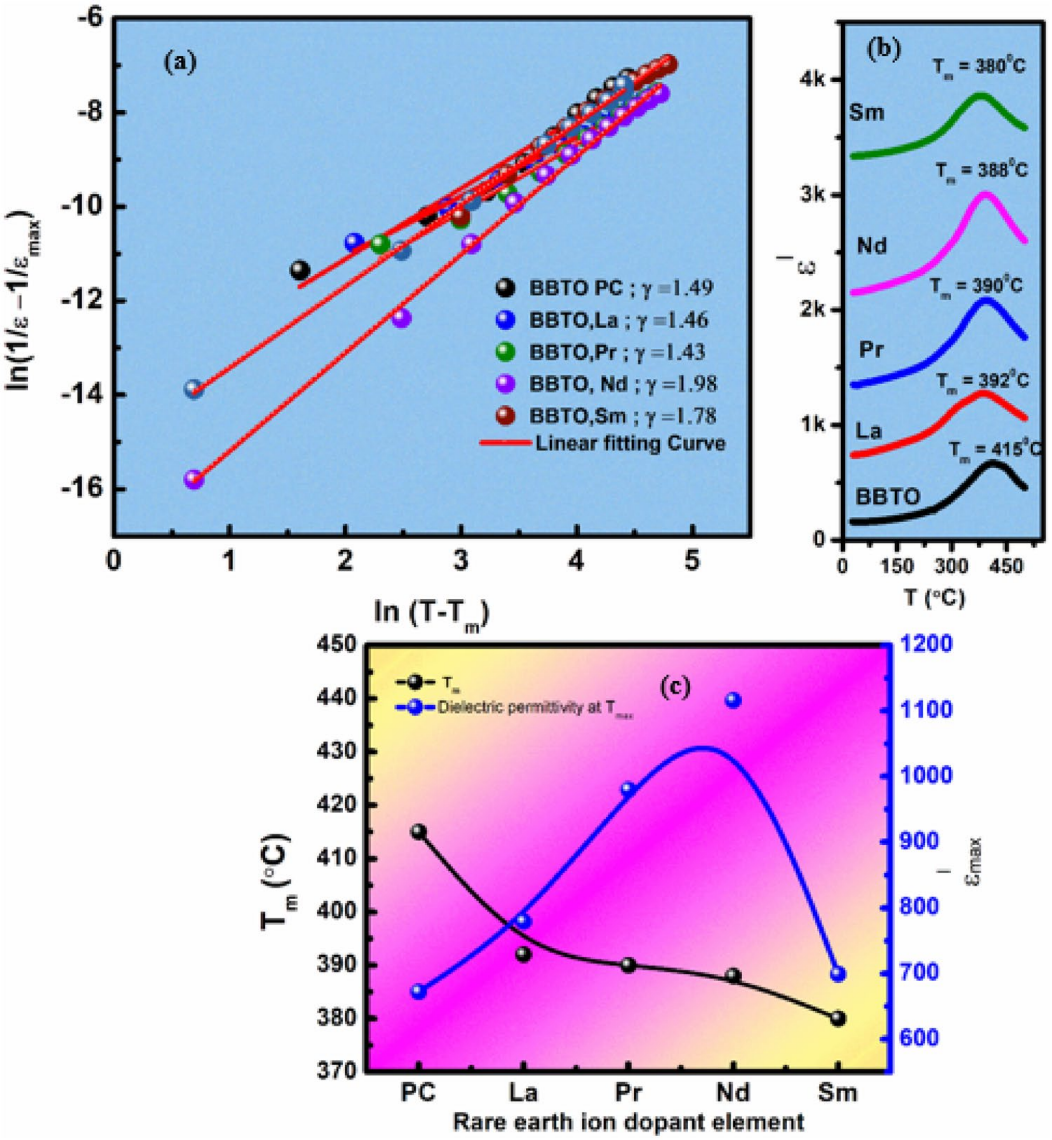
Modified Curie law fitting curves: (**a**) Variation of ln ($\frac{1}{{\varepsilon }^{l}}-\frac{1}{{\varepsilon }_{max}^{l}}$) vs. ln (T–T_m_) @ 1 MHz frequency of RE-substituted BaBi_4−x_RE_x_Ti_4_O_15_ (x = 0.10, RE = La, Pr, Nd, and Sm) ceramics, (**b**) A clear distinct ferroelectric phase transitions (T_m_) of BBTO and substituted BBTO ceramics @ 1 MHz frequency point. (**c**) The plots represent variation of T_m_ and ${\varepsilon }_{max}^{1}$ with increasing ionic radii of substituted RE-element.

Here ${\varepsilon }_{max}^{l}$ is the maximum dielectric constant at T_m_, C is Curie constant, T is the absolute temperature and γ is the degree of diffuseness (2 ≤ $\gamma$  ≤ 1). The diffuseness constant $\gamma =1$ is for normal ferroelectric and $\gamma$ = 2 for relaxor ferroelectrics^41,48^. In the present study, the diffuse relaxor activity can be systematically enlightened by fitting dielectric plot ln ($\frac{1}{{\upvarepsilon }^{\mathrm{l}}}-\frac{1}{{\upvarepsilon }_{\mathrm{max}}^{\mathrm{l}}}$) vs. ln (T − T_m_) @ 1 MHz frequency as shown in Fig. 8a ^41^. The slope of the curve represents the diffuseness constant ($\upgamma$), as displayed at the inset of Fig. 8a. The obtained $\upgamma$ values are in the range between 1.72 and 1.98, emphasizing more diffuse activity in RE^3+^ ion-substituted BBTO ceramics. The observed diffuse relaxor property due to random redistribution of number of cations at the A-site (i.e., RE^3+^, Bi^3+^ and Ba^2+^ions) in RE^3+^ ion-substituted BBTO ceramics. The larger number of cationic redistributions at A-site can easily produce a loosely structure with a large c-axis, which may cause the short-range ordering with increased degree of diffusion^44,45^.

Figure 8b illustrates a clear distinct phase transitions (T_m_) at 415, 392, 390, 388, 380 °C of pure BBTO and La^3+^, Pr^3+^, Nd^3+^ and Sm^3+^ion-substituted BBTO ceramics, respectively for the frequency of 1 MHz. The Curie temperature (T_m_) decreases towards the lower temperature region with the increase of ionic radii for RE^3+^ dopants in BBTO ceramics. The noted dielectric maxima (${\varepsilon }_{m}^{1}$) at T_m_ increases for all RE-substituted BBTO ceramics as shown in Fig. 8c, except for Sm^3+^ ion-substituted BBTO ceramics^45^. In general, the Curie temperature (T_m_) in ferroelectrics always depends on the nature (electronegativity) and amount of substituent leading to structural distortion. Shimakawa et al. suggests that the Curie temperature (T_m_) of layered Aurivillius materials for lattice distortion in the pseudo-perovskite interior is inversely related to tolerance factor (*t*)^46,47^. As the average tolerance factor decreases in RE^3+^ ion-substituted BBTO ceramics, due to decrease ionic radii of substituents at A-site in layered Aurivillius ceramics. Therefore, the reduction of structural distortion leads to a decrease in orthorhombicity indicating crystal structure symmetry increases continuously. This could lead to a decrease in the ferroelectric transition temperature (T_m_) in RE^3+^ ion-substituted BBTO ceramics^48–50^.

**Figure 9:**
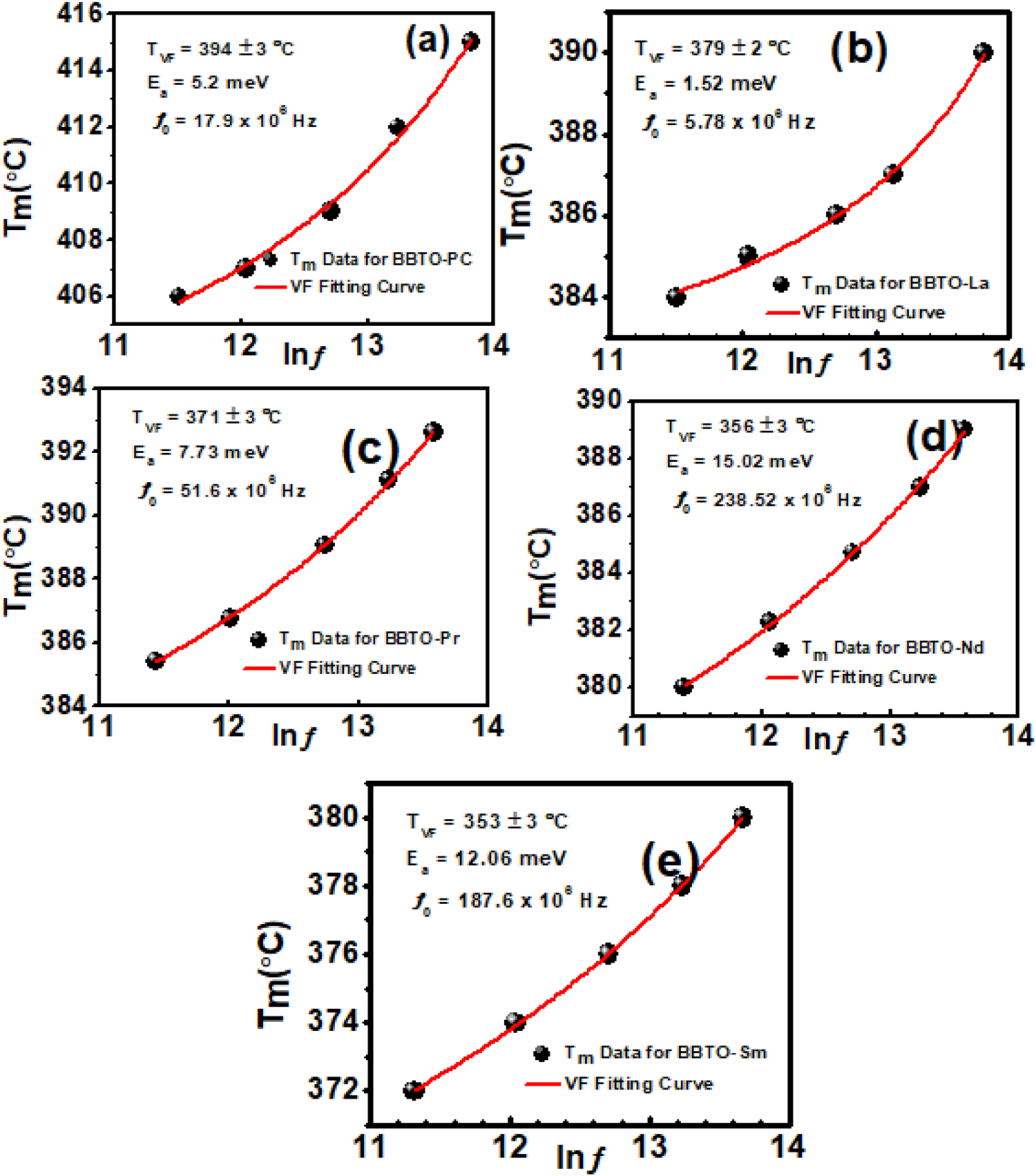
A nonlinear Vogel–Fulcher law curve fitting of RE-substituted BaBi_4−x_RE_x_Ti_4_O_15_ (x = 0.10, RE = La, Pr, Nd, and Sm) ceramics: (**a**) pure $\mathrm{BBTO}$, (**b**) BBTO-La, (**c**) BBTO-Pr, (**d**) BBTO-Nd, and (**e**) BBTO-Sm, respectively. Inset of the Figure represents the fitting parameters i.e., E_a_, T_VF_ and f_m_ of the corresponding ceramics.

In present study, it was noticed a strong frequency dependence in dielectric permittivity along with dielectric loss, which is the characteristics of relaxor ferroelectrics. Considering this point, we explained relaxor property with help of nonlinear Vogel–Fulcher (VF) relation^51^

3 $$f={f}_{o}\mathrm{exp}\left(\frac{-{E}_{a}}{{k}_{B}\left({T}_{m}-{T}_{VF}\right)}\right),$$

where *f* is the measured frequency, ${f}_{o}$ is the cut off frequency at free relaxation time (Debye frequency), *E*_*a*_ is the activation energy describing the relaxation process, k_B_ is the Boltzmann constant and T_VF_ is the freezing temperature at which all relaxation times (τ) become infinitely broaden. Figure 9a–e depicts the frequency dependent dielectric temperature (T_m_ (°C)) vs. ln $f$ as well fitted with nonlinear VF relation for pure and RE^3+^ ion-substituted BBTO ceramics. Furthermore, the dielectric loss spectra also exhibit frequency dependent temperature maxima (${T}_{m}^{ll}$), which is slightly lower than dielectric permittivity (T_m_) peaks (supplementary information inset of Supplementary Fig. S2a–e). In contrast, the frequency dependent dielectric loss is scarcely observed in barium modified Aurivillius ceramics that do not obey VF relation ^49,50^. The relaxor behaviour in BaBi_4_Ti_4_O_15_ and in RE^3+^ ion-substituted BaBi_4−x_RE_x_Ti_4_O_15_ ceramics can be explained in terms of formation of anti-site defects at A-site because of entering Bi^3+^ ions or substitution of RE^3+^ ion, while Ba^2+^ ions are incorporated into (Bi_2_O_2_)^2+^ layers. Therefore, the local inhomogeneous distribution of barium, RE^3+^ ions into (Bi_2_O_2_)^2+^ layers and charge imbalance due to anti-site defects at A-site ions lead to the formation of relaxor activity^49,52^.

The parameters obtained after fitting such as T_VF_, *E*_*a*_ and ${f}_{o}$ are presented in the inset of Fig. 9a–e, which are in well agreement with the previous reports^53,54^. The calculated activation energies are in the range between 1 to 12 meV, and relaxation time (${\tau }_{0}$= $\frac{1}{{f}_{o}}$) from 10^–6^ to 10^–8^ s, respectively for all RE^3+^ ion-substituted BBTO ceramics. The obtained activation energy and relaxation time are attributed to positional disorder of cations at A or B sites of perovskite blocks interrupting the evolution of long rage polar ordering. This could lead to the formation of a small polaron hopping closer to phase transition, which are supported in dc conductivity studies.

### Complex conductivity spectroscopy

#### Frequency dependent conductivity

**Figure 10:**
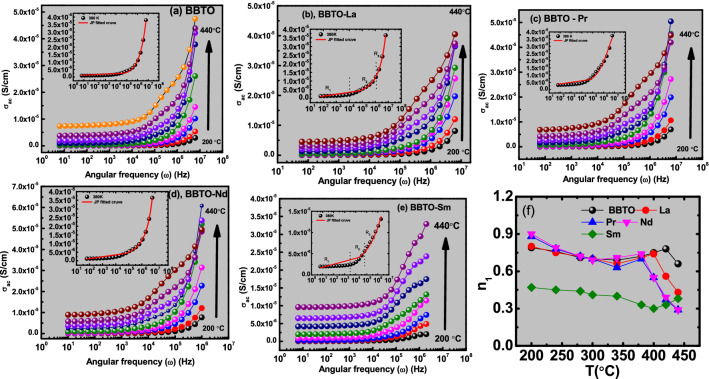
Plot represents the Jonscher’s Power law fitting curves between ac-conductivity (${\sigma }_{ac}$) vs. angular frequency ($\omega$) in temperature range from 200 to 450 °C of (**a**) pure $\mathrm{BBTO}$, (**b**) BBTO-La, (**c**) BBTO-Pr, (**d**) BBTO-Nd, and (**e**) BBTO-Sm ceramics, respectively. (**f**) Plot of exponent n_1_ vs. temperature.

Temperature dependent electrical properties and relaxations kinetics with the correlation of conductivity in pure BBTO and RE^3+^ ion-substituted BBTO ceramics was investigated by expending ac conductivity study. Figure 10a–e exhibits the variation ac conductivity (${\sigma }_{ac}$) as function of angular frequency ($\omega$) in the temperature range from 200 to 450 °C. The ac conductivity value was calculated from empirical dielectric constant and dielectric loss relation as follows^55–57^:

4 $${\sigma }_{ac}={\omega \varepsilon }_{0}{\varepsilon }_{r} \; \mathrm{tan \delta },$$

where ${\upvarepsilon }_{0}$ is permittivity in free space, ${\varepsilon }_{r},$ is dielectric constant and $\upomega$ is the angular frequency. The frequency dependent ac conductivity in RE-substituted BBTO ceramics obeys universal Jonscher’s Power law^58^ (Eq. 6) from 200 to 440 °C as shown in Fig. 10a–e.

5 $${\sigma }_{T}(\omega )= {\sigma }_{dc}(0)+{\sigma }_{ac}(\omega ), (\mathrm{where }\; {\sigma }_{ac}(\omega )={A}_{1}{\omega }^{{n}_{1}}+{A}_{2}{\omega }^{{n}_{2}}).$$

Therefore, total conductivity will be given by the following relation:

6 $${\sigma }_{T}(\omega )= {\sigma }_{dc}(0)+{A}_{1}{\omega }^{{n}_{1}}+{A}_{2}{\omega }^{{n}_{2}},$$

where ${\sigma }_{ac}\left(\omega \right)$ represents ac-conductivity, ${\sigma }_{dc}\left(0\right)$ represents dc conductivity (dc plateau regions in Fig. 10a–e), and the coefficients are A_1_, A_2_ and exponents n_1_ and n_2_ represented from the slopes of corresponding regions. The parameter n (0 $\le$ n_1_, n_2_ $\le$ 1) is independent of frequency, but it depends on temperature and material’s intrinsic property. Here, ${\sigma }_{ac}(\omega )={A}_{1}{\omega }^{{n}_{1}}$ +${A}_{2}{\omega }^{{n}_{2}}$ is frequency dependent that characterizes frequency dispersions. The experimental data (closed circles) points are well agreed with fitted power law curves (red solid curve shown in inset of Fig. 10a–e). The obtained dc conductivity (${\sigma }_{dc}\left(0\right))$ values are tabulated in Table 4 for RE^3+^ substituent in BBTO ceramics. The ac conductivity strongly becomes frequency dependent at temperature below 340 °C for BBTO and RE-substituted BBTO ceramics. The temperature above 340 °C, dc conductivity for (ω) ≤ 10^5^ Hz provides the frequency independent plateau region whereas for (ω) ≥ 10^5^ Hz a frequency dependent conductivity is obtained in BBTO ceramics. The frequency independent region for (ω) ≤ 10^4^ Hz in RE^3+^ ion-substituted BBTO ceramics, indicating the length of plateau region decreases. At low frequency and low temperature regions, the strong frequency dispersion is observed in all samples. These are represented by R_2_ and R_3_ regions as shown in inset of Fig. 10a–e^56–58^.

The observed frequency independent conductivity (${\sigma }_{dc}$) at high temperature for (ω) ≥ 10^4^ Hz can be explained with help of jump relaxation model^59^. In BBTO and RE^3+^ ion-substituted BBTO ceramics, there might have defective ions due to oxygen vacancies. These ions successfully hop to its neighbourhood vacant sites, but these may take long time. The successive hoping of ions results in a long-range translational motion. This leads to dc conductivity in RE^3+^ ion-substituted BBTO Aurivillius oxides. The frequency dependent conductivity at high temperature can be explained with the help of Correlated Barrier Hopping (CBH) model, where exponent n_1_ decreases with the increase of temperature as shown in Fig. 10f. In CBH model, a mobile charge carrier hops to a new site from its original position; then it remains in a state of displacement between two potential energy maxima. After that, the neighbour ions relaxed with respect to new sites. Such a dominated forward or backward hopping of ion charge carriers or thermally activated oxygen vacancies could lead to more dispersive conductive nature at high frequency region^60,61^.

**Figure 11:**
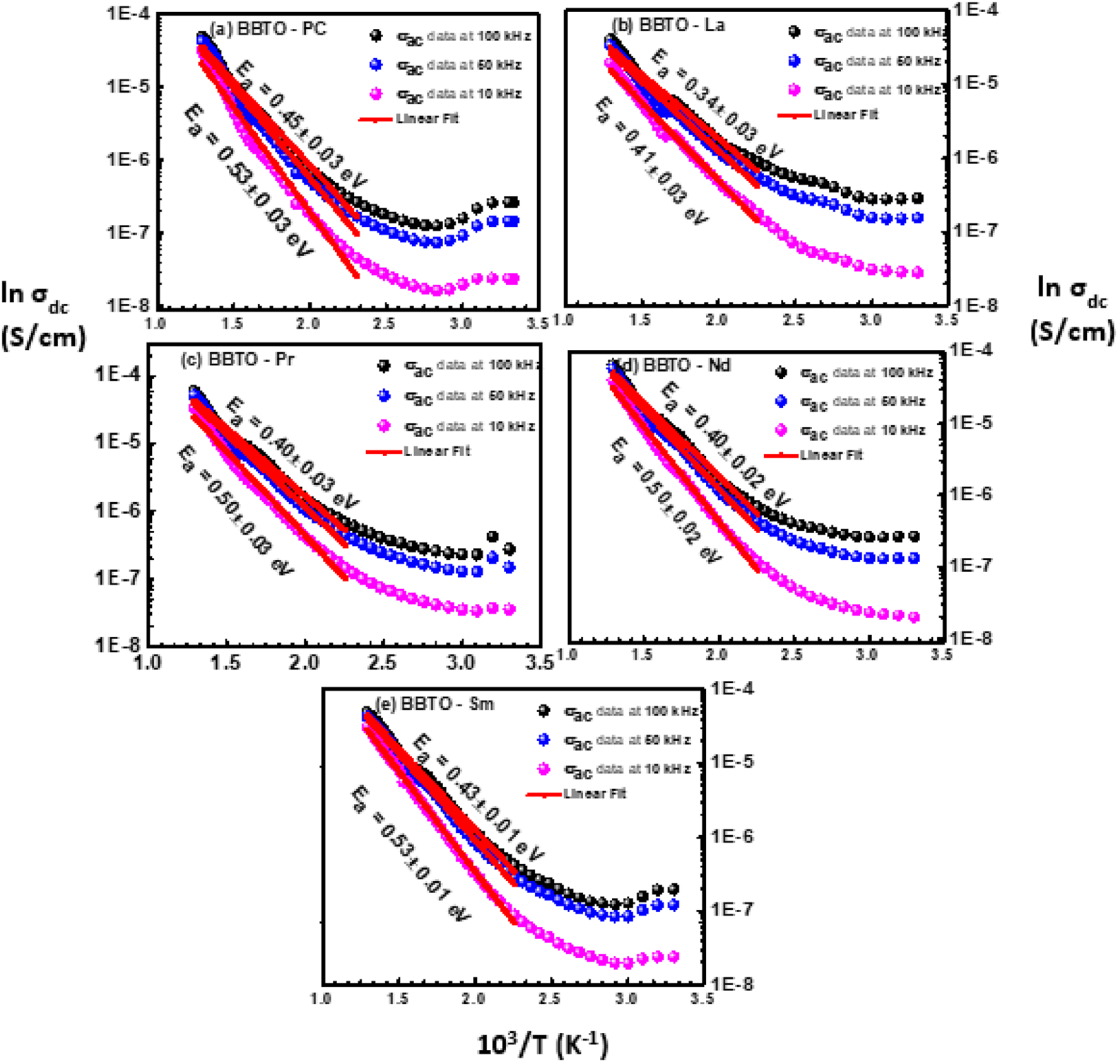
Arrhenius peak fitting using ln(${\sigma }_{dc}$) vs. 1/T graphs at fixed frequency of RE-substituted BaBi_4−x_RE_x_Ti_4_O_15_ (x = 0.10, RE = La, Pr, Nd, and Sm) ceramics: (**a**) pure $\mathrm{BBTO}$, (**b**) BBTO-La, (**c**) BBTO-Pr, (**d**) BBTO-Nd, and (**e**) BBTO-Sm ceramics, respectively.

The dc conductivity ${\sigma }_{dc}\left(0\right)$ data can be theoretically predicted from the extrapolation of ac conductivity vs. frequency (ω) graphs (Fig. 11) with the help of power law fitting. Thus, the temperature dependent exponent (n_2_) and ${\sigma }_{dc}\left(0\right)$ values are listed in Table 4. The dc-conductivity increases with increase of temperature for all ceramics, which indicates a negative temperature co-efficient of resistance (NTCR) behaviour. This is the characteristics of semiconductor nature of RE^3+^ ion-substituted BBTO Aurivillius ceramics. On contrary to pure BBTO-sample, the RE^3+^ ion-substituted BBTO Aurivillius ceramics show decrement in dc conductivity values (Table 4). The decrease in conductivity for RE^3+^ ion-substituted BBTO ceramics might be due to low space-charge, low domain pinning and a smaller number of vacancies accumulated at the interfaces^62,63^.

Temperature dependent exponents (n_1_ and n_2_) also illustrate the physical significance to charge carriers either in motion or in localized. When n < 1, it indicates the motion consists of translational like with sudden hopping, whereas n > 1 involves to localized hopping without leaving vicinity of lattice site^64^. Based on frequency and temperature dependence of exponent (n), an internal hopping mechanism has been anticipated. An increasing trend of exponent (n) as a function of temperature is attributed to small polaron hopping, whilst the reverse trend as a function of temperature corresponds to large polaron hopping. The frequency at which the changes of exponent value taken place is known as hopping frequency of the polarons (w_p_), which is a function of temperature^64^. Here, the obtained exponent (n_1_) values are less than 1 for all BBTO ceramics as shown in Fig. 10f. This indicates translation motion of charge carriers within the ceramics. The similar trend is also noted in variation of n_2_ with respect of temperature at high frequency region (0.85 $\le$ n_2_ $\le$ 1.05). In addition to the above, the value of n_1_ is found to be decreased with increasing temperature (≤ 370 °C). This exponent value increases up to 420 °C and starts to decrease with increasing temperature above 420 °C for all RE^3+^ ion-substituted BBTO Aurivillius ceramics. Therefore, it can be concluded that the conduction mechanism arises mainly due to the short-range translation hopping via large polaron (or double ionized oxygen ions) at T ≤ 370 °C. Then, the conduction process is followed by small polaron hopping near Curie temperature region (370 °C ≤ T ≤ 420 °C). Furthermore, above paraelectric region (T > 420 °C), the large polaron hopping mechanism was stabilised. The RE^3+^ ion-substituted BBTO ceramics also exhibit similar translational conduction mechanism near T ≤ 340 °C region due to large polaron hopping via single ionized oxygens. Then small polaron hopping was established in the region from 350 to 400 °C. Worthwhile, the observed small polaron hoping at the temperature region is well matched with ferroelectric phase transition temperature (T_m_) positions of BBTO and RE-substituted BBTO Aurivillius compounds^65^.

#### Temperature dependent dc conductivity

We have studied the dc-conductivity with variation of temperature to investigate the origin of charge carriers or defect dipoles/oxygen ion vacancies, and its associated relaxation mechanism. Figure 11a–e shows the variation of dc-conductivity with temperature (log σ_dc_ vs. 10^3^/T) in pure BBTO and RE^3+^ ion-substituted BBTO ceramics. Here, the activation energy was calculated by using Arrhenius relation (Eq. 7) from the slope of the solid lines as shown in Fig. 11a–e.

7 $${\sigma }_{dc}={\sigma }_{0}exp \left(\frac{{E}_{a}}{{k}_{B}T}\right),$$

Here, ${\sigma }_{dc}$ indicates the dc conductivity, *E*_*a*_ the activation energy and *k*_*B*_ the Boltzmann constant and obtained values of activation energy are displayed in the corresponding Fig. 11a–e. The obtained activation energy values are to be 0.85 eV for pure BBTO and 0.45, 0.55, 0.60 and 0.65 eV for La^3+^, Pr^3+^, Nd^3+^ and Sm^3+^ ion-substituted BBTO ceramics, respectively. In recent reports on Ba_1−x_Sr_x_Bi_4_T_4_O_15_ and SrBi_2_Ta_2_O_9_ ceramics, the energies for diffusion of doubly ionized oxygen vacancies are in the order of 0.87–1.4 eV^66–68^. Additionally, in bismuth layered perovskite oxides for long or localized migration of doubly ionized oxygen vacancies have activation energies in the order of 1 eV^69^.

In the present work, the obtained activation energy *E*_*a*_ = 0.85 eV for pure BBTO ceramics is in well agreement with previously reported value. Therefore, the conduction mechanism can be attributed to the long-range motion of doubly ionized oxygen vacancies and relaxation due to the short-range hopping of doubly ionized oxygen vacancies in pure BBTO ceramics^69,70^. While for RE^3+^ ion-substituted BBTO ceramics, the obtained activation energies approximately 0.45–0.65 eV are in well agreement with the energies for diffusion of single ionized oxygen vacancies in layered perovskite oxides. The filled 4f orbital of the highly insulating rare earth ions (RE^3+^) substituted at Bi site may suppress the volatilization of bismuth. As a result, a decrease in oxygen vacancies and a reduction of defect dipole charge carrier concentration might be taken place in RE-substituted BBTO ceramics. This results in slight decrement in conductivity of RE-substituted BBTO ceramics as compared to pure BBTO ceramics. Henceforth, the conduction and dielectric relaxation in RE^3+^ ion-substituted BBTO ceramics might be due to singly ionized oxygen vacancies. The formation of complex defects or defect in the layered Aurivillius bismuth ceramics can be explained as follows. Generally, in layered Aurivillius bismuth perovskite oxides, during the high temperature sintering process charge defect or complex vacancies are formed in (Bi_2_O_2_)^2+^ due to bismuth (Bi_2_O_3_) volatilization^49,70–72^. The formation of oxygen vacancies in Bi_2_O_3_ oxide can be explained as per the given relation:

8 $${\mathrm{Bi}}_{2}{\mathrm{O }}_{3}\to 2\mathrm{Bi }+\frac{3}{2}{O}_{2} \hspace{0.17em}+\hspace{0.17em}2 {V}_{Bi}^{"} + 3 V_{{\ddot{O}}} .$$

As a result, the formation of either singly ionized or doubly ionized oxygen led to trap electrons according to the relation:

$${V}_{O}= V_{{\ddot{O}}}+{e}^{//},$$

9 $${V}_{O}=V_{{\ddot{O}}}+{e}^{/},$$

where $V_{{\ddot{O}}}$ and $V_{{\ddot{O}}}$ are singly and doubly ionized oxygen vacancies, respectively.

### Ferroelectric properties

**Figure 12:**
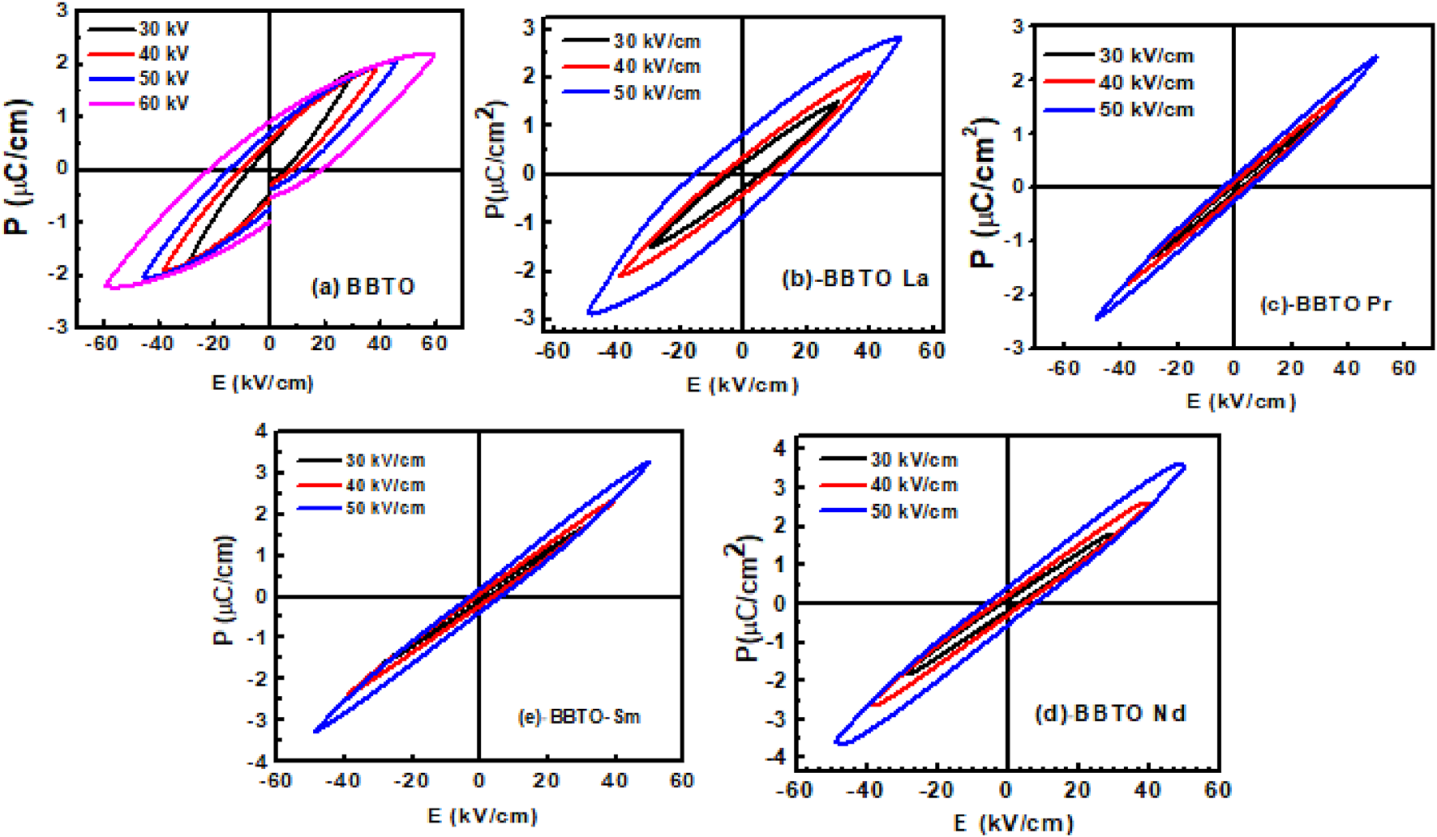
(**a**–**e**) Room temperature P–E hysteresis loops of pure BaBi_4_Ti_4_O_15_ and RE-substituted BaBi_4−x_RE_x_Ti_4_O_15_ (x = 0.10, RE = La, Pr, Nd, and Sm) ceramics.

Figure 12a–e exhibits the room temperature polarization–electric field (P–E) hysteresis loops of RE^3+^ ion-substituted BBTO Aurivillius compounds at a constant loop frequency of 50 Hz under the applied field strengths of 30, 40, and 50 kV/cm, respectively. The measured saturation and remnant polarization values are P_s_ = 2.16 μC/cm^2^ and P_r_ = 0.90 μC/cm^2^ at a maximum field of 60 kV/cm for pure BBTO ceramics. These values are in well agreement with previous reports^30,35^. With the comparison of above, the substitution of RE-element with decrease of ionic radii in BBTO ceramics exhibits the slight decrement in P_r_ values, then it starts to increase in P_s_ values as observed. Furthermore, a sharp decrement in P_s_ and E_c_ values with the substitution of RE^3+^ ions in BBTO ceramics were observed. The similar trend was also detected in T_m_ vs. composition of RE^3+^ ionic element plot (Fig. 8c). The basic motive behind this abstraction is a slight decrease in grain size for RE^3+^ ion substitutions. This could be the rate of less diffusivity during sintering process as notified in surface morphology studies^24,30^. In addition to that, the lattice parameters ($a,b$) decrease for RE^3+^ ion substitution in BBTO ceramics. In accompanying with the unit cell volume, the orthorhombic structure stress ($a/b$) decreases for La^3+^, and Pr^3+^ ion substitution, then a slight increment of the stress, was noticed for Nd^3+^, and Sm^3+^ ion substitution^20,29^. The orthorhombic structure stress could be directly associated with the degree of lattice distortion of pseudo-perovskite including BO_6_ oxygen octahedral rotation and tilting. This may be slightly changed with RE^3+^ ion substitution in BBTO ceramics. Henceforth, it was noticed a decrease in P_r_ values and the slim asymmetric P–E loops. On the other hand, the Nd^3+^ ion-substituted BBTO sample provides a thin sharp plate-like grains for specified grain growth significantly higher in the direction perpendicular to the *c*-axis. Therefore, a slight variation in P–E loops for Nd^3+^ ion- substituted BBTO ceramics was detected^59^. The study on slim asymmetric ferroelectric loops with RE-substituents in BBTO ceramics indicate more relaxor ferroelectric nature. Thus, all the observed parameters for electrical properties: T_m_, ε_r_(T), σ_ac_, E_a_, 2P_r_, tolerance factor, γ, ΔT_relax_ are summarised in Table 5 for all the BBTO ceramics.

## Conclusions

An attempt has been undertaken to synthesize RE-substituted BBTO Aurivillius ceramics using the solid state reaction method. The quantitative phase contribution and coexistence of dual phase in RE-substituted BBTO ceramics were established by XRD analyses. The randomly oriented grains with plate-like morphology, chemical composition, and elemental incorporation of RE^3+^ ionic elements in BBTO ceramics indicated the purity of bismuth layered Aurivillius ceramics. A shifting in T_m_ towards the low temperature region for RE-substituted BBTO ceramics was confirmed by temperature dependent dielectric studies. The inherent relaxor nature was explained using modified Curie–Weiss law along with Vogel–Fulcher relation. The ac conductivity study concluded the conduction mechanism assisted by large polaron hopping well below and above the T_m_ and small polaron hopping near the T_m_ region for all the ceramics. The temperature dependent conductivity study demonstrated the conduction mechanism attributed to the long-range motion of doubly ionized oxygen vacancies whereas the relaxation mechanism followed by the short-range hopping of doubly ionized oxygen vacancies in pure BBTO ceramics. Furthermore, in RE^3+^ ion-substituted BBTO ceramics, the conduction mechanism due to diffusion of single ionized oxygen vacancies was obtained. Finally, the room temperature ferroelectric study demonstrated a well-shaped ferroelectric loop for parent compound, whereas RE^3+^ ion-substituted BBTO ceramics provided the slim asymmetric ferroelectric character. Therefore, the diffuse relaxor along with slim asymmetric polarization properties in RE^3+^ ion-substituted BBTO ceramics may provide large energy storage, and high-temperature piezoelectric sensor applications.

## Supplementary Information

Supplementary Figures.
