# Supplementary material for: Fortified relaxor ferroelectricity of rare earth substituted 4-layered BaBi3.9RE0.1Ti4O15 (RE = La, Pr, Nd, and Sm) Aurivillius compounds
Source: Sci Rep. 2022 Oct 3;12:16508. doi: 10.1038/s41598-022-18855-9 (PMC9529884; doi:10.1038/s41598-022-18855-9)
Supplement: Supplementary file 1 — Supplementary Figures. [file 41598_2022_18855_MOESM1_ESM.docx]

**Fortified Relaxor Ferroelectricity of Rare Earth Substituted 4-layered BaBi_3.9_RE_0.1_Ti_4_O_15_ (RE = La, Pr, Nd, and Sm) Aurivillius Compounds**

Tirupathi Patri^1^, Avijit Ghosh^2^, M. L. V. Mahesh^,3^, P.D. Babu^4^, S. K. Mandal^5^ and M. N. Singh^6^

^1^Department of Physics, Rajiv Gandhi University of Knowledge Technologies, Srikakulam, Andhra Pradesh-532402, India

^2^Departments of Physics, Central University of Jharkhand, Ranchi, Jharkhand-835205, India

^3^Defence Metallurgical Research Laboratory, Kanchan Bagh, Hyderabad, Telangana-500066, India

^4^UGC-DAE Consortium for Scientific Research, Mumbai Center, BARC, Mumbai-400085, India

^5^Surface Physics and Materials Science Division, Saha Institute of Nuclear Physics Kolkata, Sector-1, AF Block, Bidhannagar, Kolkata-700064, India

^6^HXAL Synchrotrons Utilization Section, Raja Ramanna Centre for Advanced Technology Indore-452013, India


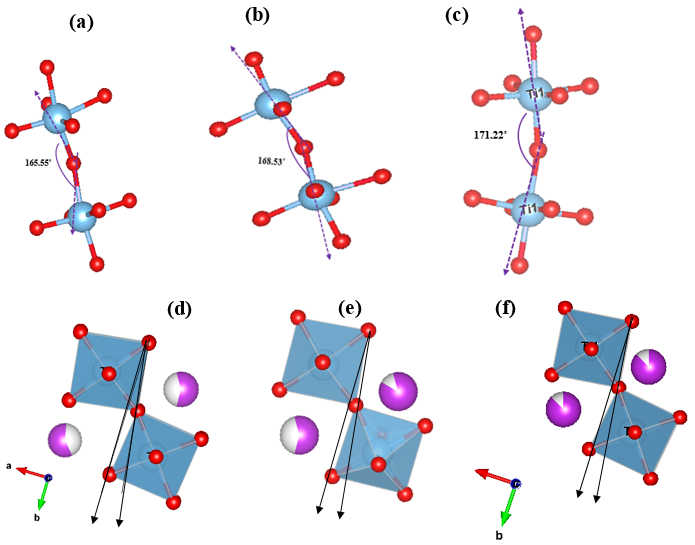


**Figure-S1:** The observations of crystal structure and atomic displacement re-ordering from Rietveld refinements on XRD patterns: **(a-c)** distortion of *B*O_6_ octahedra from the *c*-axis, and **(d-f)** tilt angle of rotation in *BO_6_* octahedra projected along the *c*-axis for BBTO, BBTO-Pr, and BBTO-Sm ceramics, respectively.


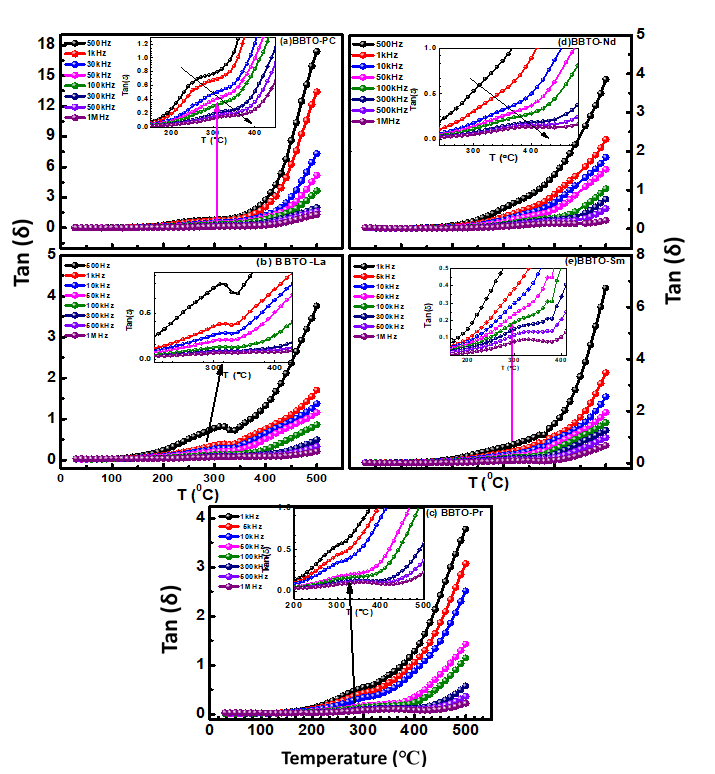


**Figure-S2:** Variation of dielectric loss with temperature (tan$\delta$ vs. T) in the temperature interval of 30 to 500 °C with fixed frequency of pure BaBi_4_Ti_4_O_15_ and RE-substituted BaBi_4-x_RE_x_Ti_4_O_15_ (x = 0.10, RE = La, Pr, Nd, and Sm) ceramics: (**a**) pure $\mathrm{BBTO}$, (**b**) La-substituted BBTO, (**c**) Pr-substituted BBTO, **(d)** Nd-substituted BBTO, and (**e**) Sm-substituted BBTO ceramics, respectively.
